# A systematic approach to RNA-associated motif discovery

**DOI:** 10.1186/s12864-018-4528-x

**Published:** 2018-02-14

**Authors:** Tian Gao, Jiang Shu, Juan Cui

**Affiliations:** Systems Biology and Biomedical Informatics (SBBI) Laboratory, Department of Computer Science and Engineering, University of Nebraska-Lincoln, Lincoln, NE 68588 USA

**Keywords:** Motif finding, Short sequences, Exosomes, microRNAs, Exosomal RNAs, Graph algorithms

## Abstract

**Background:**

Sequencing-based large screening of RNA-protein and RNA-RNA interactions has enabled the mechanistic study of post-transcriptional RNA processing and sorting, including exosome-mediated RNA secretion. The downstream analysis of RNA binding sites has encouraged the investigation of novel sequence motifs, which resulted in exceptional new challenges for identifying motifs from very short sequences (e.g., small non-coding RNAs or truncated messenger RNAs), where conventional methods tend to be ineffective. To address these challenges, we propose a novel motif-finding method and validate it on a wide range of RNA applications.

**Results:**

We first perform motif analysis on microRNAs and longer RNA fragments from various cellular and exosomal sources, and then validate our prediction through literature search and experimental test. For example, a 4 bp-long motif, GUUG, was detected to be responsible for microRNA loading in exosomes involved in human colon cancer (SW620). Additional performance comparisons in various case studies have shown that this new approach outperforms several existing state-of-the-art methods in detecting motifs with exceptional high coverage and explicitness.

**Conclusions:**

In this work, we have demonstrated the promising performance of a new motif discovery approach that is particularly effective in current RNA applications. Important discoveries resulting from this work include the identification of possible RNA-loading motifs in a variety of exosomes, as well as novel insights in sequence features of RNA cargos, i.e., short non-coding RNAs and messenger RNAs may share similar loading mechanism into exosomes. This method has been implemented and deployed as a new webserver named MDS^2^ which is accessible at http://sbbi-panda.unl.edu/MDS2/, along with a standalone package available for download at https://github.com/sbbi/MDS2.

**Electronic supplementary material:**

The online version of this article (10.1186/s12864-018-4528-x) contains supplementary material, which is available to authorized users.

## Background

Motif finding has been a long-standing problem in DNA sequence analysis where numerous tools have been developed to identify cis-regulatory elements and to unravel mechanisms of gene expression regulation [1]. Those conventional algorithms were designed to identify statistically significantly overrepresented sequence patterns based on two major resources: (1) the promoter regions of co-expressed genes, which are normally over 1000 nt long; (2) targeted binding regions (less than 300 nt in length) of the known transcription factor (TF), which typically captured by sequencing technology such as Chromatin Immunoprecipitation Sequencing (ChIP-Seq) [2, 3]. Up to now, similar discovery of regulatory binding sites has been dramatically revolutionized by recent emergence of other new technologies. For example, RNA Immunoprecipitation Sequencing (RIP-Seq) is designed to explore the protein binding sites on RNA; Photoactivatable Ribonucleoside-Enhanced Crosslinking and Immunoprecipitation (PAR-CLIP) reveals binding sites of proteins, e.g., Human Argonaute (AGO) proteins, on messenger RNAs (mRNAs) [4–6]; and the crosslinking, ligation, and sequencing of hybrids (CLASH) method is designed to capture microRNA (miRNA)-mRNA interactions [7, 8]. To investigate the (post-)transcriptional regulation from different mechanistic perspectives, each of those sequencing analyses has warranted the revisit of motif finding that remains to be challenging due to the intensive computation and high-order of complexity on pattern search, as well as some new difficulties. For instance, different regulatory mechanisms imply different types of motifs. The TF-binding DNA motif is usually longer than 10 bps according to the annotated TF binding motifs from ENCODE project [9] and JASPAR database [10]. On the other hands, the binding sites in protein-RNA interaction can be as short as 3-5 bps [11–13], e.g., AGO protein binds to a mature miRNA sequence within less than 4pb long sites, typically at position 8–11 of miRNA [11]. Furthermore, miRNA-mRNA interaction sites in human can be discontinuous, showing separate complementary regions [8, 14]. Given such differences, some conventional motif finding methods (such as MEME and COSMO [15, 16]) may not be appropriate since they were not originally designed to detect short and discontinuous motifs based on possibly large sets of short RNA sequences such as miRNAs and mRNA fragments.

Very recently, exosome-mediated intercellular communication has drawn substantially increased research attention. As important mediators, exosomes transfer various types of cargos from donor cells to recipient cells, including miRNAs, truncated mRNAs, and other non-coding RNAs [17–22]. Accumulating evidence has shown the association between exosome-delivered miRNAs and developments of complex diseases such as obesity [23, 24] and cancers [25–29]. Despite the rapidly growing knowledge on exosomal RNAs’ function in acceptor cell, the process of packaging RNAs into exosomes is still largely unknown. Ohshimaa et al. found that let-7, a miRNA family with relatively high abundance in six types of cancer cell, is only detectable in extracellular exosomes of gastric cancer cell (AZ-P7a) but not others [30], which indicates cell-specific packaging mechanisms may favor some RNAs against the others. Similar observations confirmed that the content of RNA cargos in exosome is not random and the RNA loading process is highly selective [31–36]. To investigate signals that possibly guide miRNA loading, many studies have attempted to identify sequence motifs among exosomal miRNAs using de novo motif finding approaches [12, 13, 37]. For example, Villarroya-Beltri et al. predicted two motifs that might be associated with the heterogeneous nuclear ribonucleoprotein A2B1 (hnRNPA2B1)-mediated miRNA loading [12] based on 30 exosomal miRNAs using COSMO [16]. Similarly, Cha et al. found three distinct 10 bp-long motifs when using MEME [15] on exosomal miRNAs from three batches of colon cancer cells, where each motif is present only in less than 50% of the sequences [37]. Santangelo et al. detected one 6 bp-long motif pattern, [GAU][GUA][GAU][CAG][UA][GC], using Improbizer [38] on 103 exosomal miRNA sequences in murine hepatocyte 3A cells, however only GGCU is validated through cell transfection experiment [13].

More similar exosomal RNA analyses started to reveal common problems using existing motif finding methods as they tend to identify less-explicit motif patterns and overlook the possible short motifs among short sequence source (miRNAs: ~ 22 bps; partial mRNAs: ~ 100 bps). Since the number of input sequences is very limited, e.g., often less than one hundred exosomal RNAs reported in one cellular source, it may not be sufficient for EM-based methods to optimize the model and detect the best motifs through parameter tuning. On the other hands, many tools (e.g., BEEML-PBM [39], RAP [40], Inimotif [41] and AutoSeed [42]) were developed specifically for short motif prediction on protein binding microarray and (HT-)SELEX data. Since these data normally contain a large number of short sequences (over 100,000 sequence in length of 20 bp ~ 60 bp), there is an advantage of using purified sufficient input information (short and abundant). Usually these tools utilize 8-mers as seed to search for highly repetitive short motifs among the input sequences. Moreover, requirement of high-quality negative data by some tools, e.g., DREME [43], a modified version of MEME, could also be problematic. Due to our incomplete understanding of the heterogeneity of exosomal content [44] and the technical limits of detection [45], it is impractical to define a quality negative exosomal miRNA set. The significance evaluation solely depending on one positive dataset by DREME and MEME could lead to high-level false motif prediction. In addition, a few graph-based motif finding methods, such as MotifCut [46] and MotifClick [47], attempted to group similar *k*-mers to form meaningful motifs utilizing graph-based algorithms (e.g., to find the maximum-density subgraph and merge the maximum cliques). However, they usually overlook the significance evaluation of *k*-mers in order to optimize the computational process.

In this study, we propose a new method for Motif Discovery based on Short Nucleotide Sequences (MDS^2^) to overcome the aforementioned challenges. MDS^2^ is designed to conduct an unbiased search for statistically significant short motif candidates of any length among given sequences. Meanwhile, MDS^2^ optimizes the final motif pattern by balancing the sequence coverage and false detection.

## Methods

### Data acquisition

In this work, nucleotide sequences were collected from various well-established public databases and literature (Table 1). For example, a total of 1891 unique extracellular miRNAs were collected from 35 types of human and mouse cell lines and 9 types of body fluids [48–50]. An independent set of 133 literature-reported exosomal miRNAs [12, 13] are included for validation. In addition to miRNAs, other types of small RNA were collected from recently-released sequencing data, including 1064 mRNA binding sites of hsa-miR-92a-3p from CLASH data [51] and 545 AGO2 binding sites on mRNAs from PAR-CLIP data [52].

**Table 1:** Detailed statistics of the datasets in this study

Category	Content	Source	Number of cell lines	Number of unique sequences	Reference
Extracellular miRNAs	Sequences of annotated extracellular miRNAs from multiple cell lines of human and mouse^a^	ExoCarta	22	1392	[48]
		EVpedia	21	663	[49]
		Vesiclepedia	19	833	[50]
		Villarroya-Beltri et al.	1	30	[12]
		Santangelo et al.	1	103	[13]
miRNA-mRNA binding sites detected by CLASH	Sequences of hsa-miR-92a-3p binding sits on the target mRNAs	CLASH	1	1064	[8]
AGO2-mRNA binding sites detected by PAR-CLIP	Sequences of AGO2 binding sites on mRNAs	Erhard et al.	1	545	[52]

^a^Note that extracellular miRNA databases have overlapped entries, which results in a total of 1891 unique miRNAs

### Construction of a di-mer graph representing input sequences

For each group of RNA sequences, a directed di-mer graph was built to represent all given sequences. Each node represents a di-nucleotide and each directed edge connects two overlapping di-nucleotides. Therefore, a sequence, e.g., UUAAGA, will be represented by a path connecting all consecutive nodes, (UU)➔(UA)➔(AA)➔(AG)➔(GA). Each node or edge has its coverage information, including the count and the index of sequences that contain the corresponding node or edge. To narrow down the search for possible motifs, edges were removed from the graph if the coverage is less than 10% of the input sequences.

Here, a sampling process was performed to generate two sets of background sequences that facilitate the significance evaluation of each di-mer and future *k*-mer (*k* ≥ 3). One control is generated with randomly selected sequences from reference genome or RefSeq depending on the type of input nucleotide sequence (DNA or RNA), and the other contains all annotated miRNA sequences in the corresponding species (when miRNA sequences used as the input data). The procedures are summarized as follows.

**Fig. 1:**
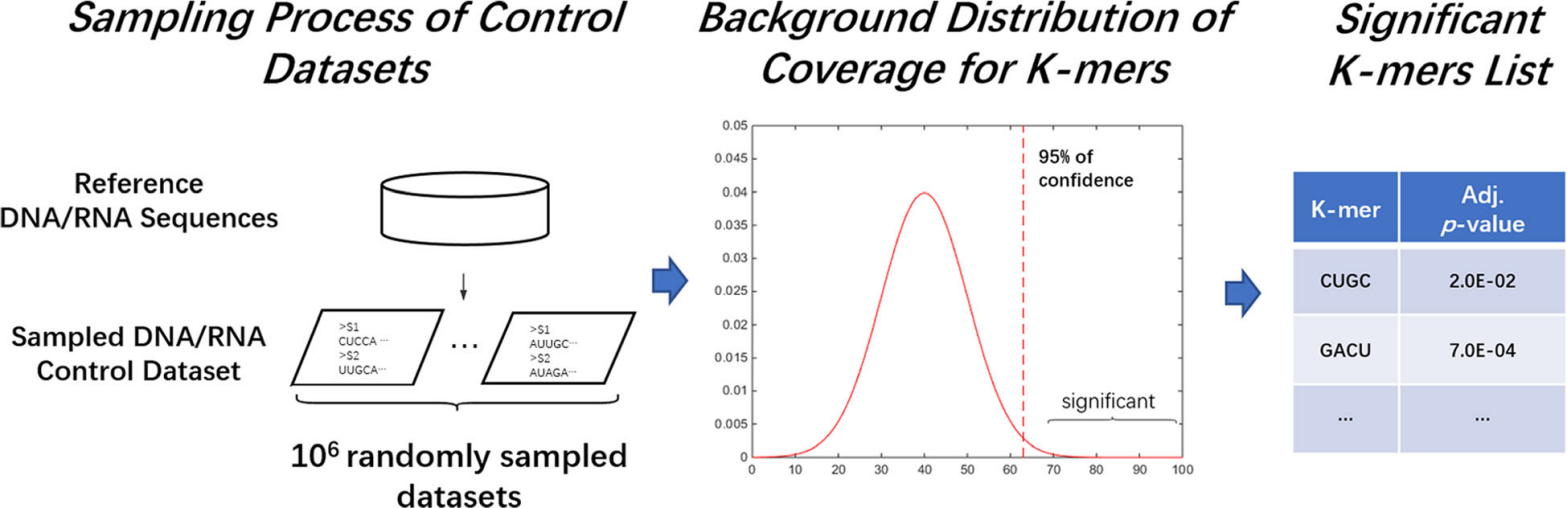
Sampling and significance evaluation on* k*-mers

Given an input size *s*, a new set of *s* sequences will be randomly generated by slicing fragments with matched length of each input sequence, from randomly selected RefSeq sequence or genome region (and miRNA sequences if applicable). The sampled sequences were then randomly shuffled. This sampling process was repeated for 10^6^ times to generate 10^6^ individual sampled control sets (Fig. 1). Next, for each di-mer, the background coverages are calculated in every sampled set, represented by a vector *C*_*set*_ = {*C*^*1*^_*set*_, *C*^*2*^_*set*_, …, *C*^*10^6*^_*set*_}. The statistical significance was evaluated by comparing input coverage *C*_*input*_ to the background *C*_*bg*_ and the raw *p*-value was computed by positioning *C*_*input*_ on the background distribution *C*_*set*_. The raw *p*-value was calculated for each di-mer as follows:

1 $$ p- value=\frac{n_{\left({C}_{set}\ge {C}_{input}\right)}}{N_{total\kern0.34em sampled\kern0.34em sets}} $$

where the denominator denotes the total number of equal-size sampled datasets (*N*), which is 10^6^ in this study; and the numerator represents the number of sampled dataset (*n*) that has a higher coverage (C_set_) of the corresponding *k*-mer than the coverage in the input dataset, *C*_*input*_.

Based on the large-scale sampling, we were able to estimate the background distribution of a certain di-mer’s coverage in the similar datasets (with the same sequence length and counts) as the input data. Thus, if the coverage of the di-mer in the input data lies in the top 5% on the background distribution, we can conclude the di-mer is significantly overpresented in the input data. The Bonferroni multiple test correction was applied to all raw *p*-values based on the number of di-mers assessed. Then, the di-mers with an adjusted *p*-value less than 0.05 were considered to be statistically significant. For miRNA tasks, the *p*-value from RNA background was the primary indicator on the significance evaluation while the miRNA background *p*-value was only used as an additional reference.

Subsequently, *k*-mers (*k* ≥ 3) can be obtained by searching for possible connecting paths. For example, the 3-mers were selected by finding all 3-node-paths in the di-mer graph. Moreover, since the edge index contains the input sequence ids, the index intersections of any two edges was obtained to decide the coverage of corresponding 3-nodes-paths. If the coverage of a 3-mer is less than 10% of total sequences, the 3-mer was filtered out. The adjusted *p*-value of each k-mer was calculated following the aforementioned procedure (Fig. 1). Only k-mers that present more than 10% of input sequences and with statistical significance (adjusted *p*-value < 0.05) were maintained for further analysis. Since underrepresented edges have been removed when the di-mer graph was built, the computational time of current search for longer fragments was reasonably controlled.

### Motif detection

**Fig. 2:**
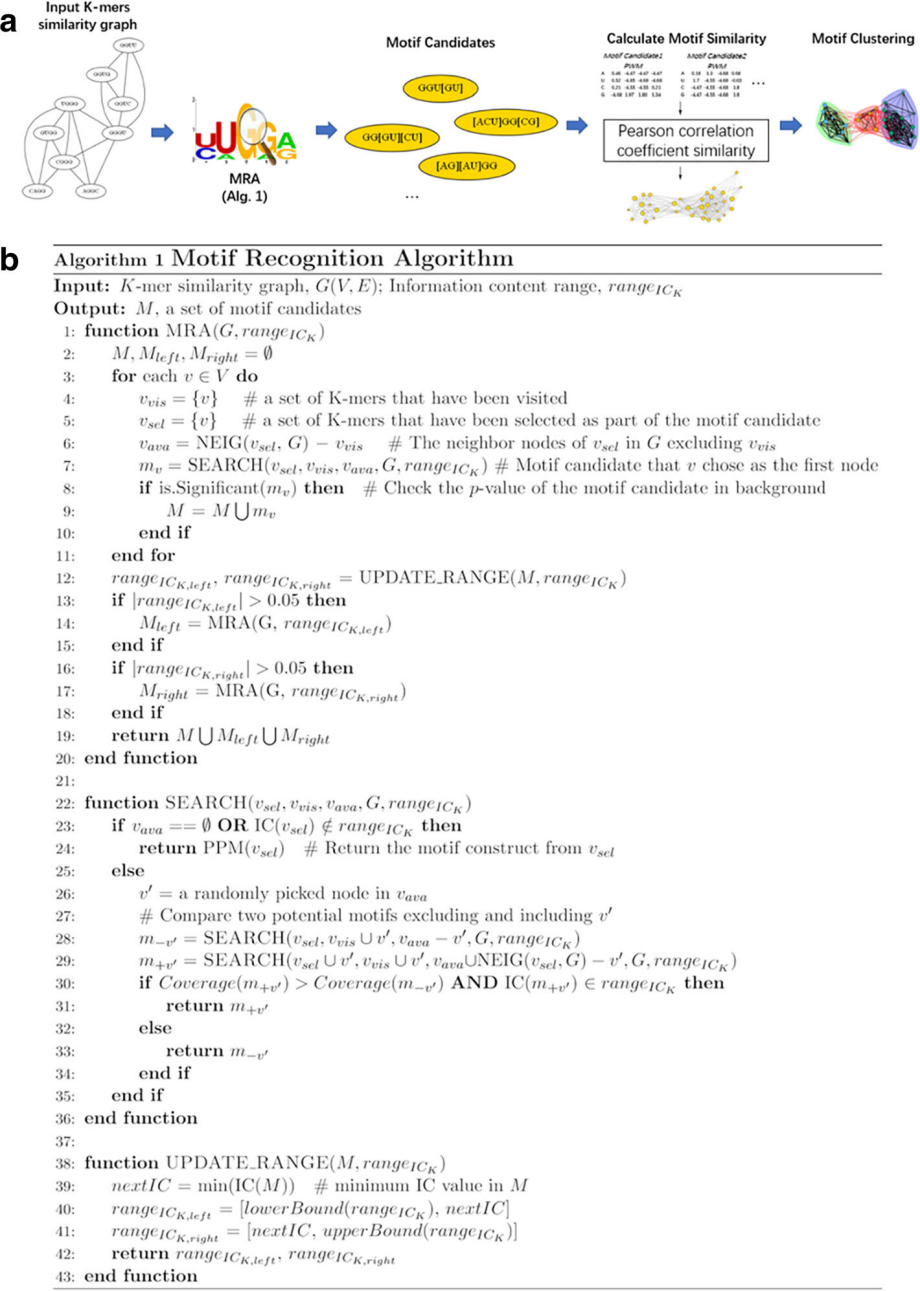
**a** Schematic workflow for motif detection based on a *k*-mer graph. **b** Description of the MRA (Motif Recognition Algorithm)

Next, a similarity graph (undirected and unweighted) was constructed (Fig. 2a) by connecting any two significant *k*-mers when they share at least (*k*-2) conserved positions, e.g., (AACG and AATT) and (ACGT and ATGG). A Motif Recognition Algorithm (MRA) was designed and implemented to identify motif patterns after merging similar *k*-mers in the similarity graph (Fig. 2b). Following the principles of the backpack problem, MRA performs the search for subsets of *k*-mers that compose optimal motifs with the highest efficacy (coverage) and different levels of explicitness (information content (IC)). It includes two sub functions: *Update_Range* and *Search*. The *Update_Range* function defines an initial range of IC and invokes the *Search* function to select a subset of *k*-mers that composes a candidate motif with highest coverage for each updated IC range. Here, IC calculation is based on Relative Entropy [53], as described in (2):

2 $$ IC(M)=\sum \limits_{k=1}^K\sum \limits_{i=1}^4P\left({z}_{ik}\right)\log \left(\frac{P\left({z}_{ik}\right)}{0.25}\right) $$

where *M* denotes a candidate motif produced by a set of *k*-mers; *Z* is the set of four nucleotides {A, U, C, G}; P(*z*_*ik*_) is the occurrence probability of a nucleotide *z*_*i*_ on the position *k* of the selected k-mer. The denominator 0.25 represents the random probability of a nucleotide occurring on a position. Thus, the relative entropy indicates a candidate’s information gain with respect to a random pattern with the same length.

Theoretically, the initial range of IC, ${range}_{IC_K}$, should be [0*k*, 0.61*k*], where the extrema represent -- the worst and best explicitness -- motifs with random nucleotides on each position (IC = 0) and motifs with conserved nucleotides on each position (IC = 0.61 k), respectively. However, we increased the lower bound to 0.125*k* (a cutoff extracted based on our heuristic analysis of the IC distribution for all collected *k*-mers) in order to reduce the computation load. Interactively, MRA changes the range of IC according to each newly-identified motif and ensure all possible candidate motifs can be assessed. Similar to *k*-mer detection, the significance evaluation was performed by comparing the coverages of the candidate motif in the input dataset and the background dataset. While the raw *p*-value of the motif candidate was calculated as described in (1), a filtering process was applied to exclude insignificant ones based on Bonferroni multiple test correction (adjusted *p*-value < 0.05).

Furthermore, to avoid high redundancy among motifs, motif similarities were evaluated. Here, TOMTOM [54] was utilized to find the optimal alignment between two motifs. The similarity is calculated by averaging the linear Pearson correlation coefficients (PCC) [55] of each position between two aligned position weight matrices (PWMs) according to the optimal offset:

3 $$ PCC\left(X,Y\right)=\frac{\sum \limits_{z\in Z}\left({X}_z-\overline{X}\right)\left({Y}_z-\overline{Y}\right)}{\sqrt{\sum \limits_{z\in Z}{\left({X}_z-\overline{X}\right)}^2\sum \limits_{z\in Z}{\left({Y}_z-\overline{Y}\right)}^2}} $$

Where *X* and *Y* denote the same column in two aligned position weight matrices (PWMs) of motifs, and $\overline{X}$ and $\overline{Y}$are the mean values of each column. *Z* is a set of four nucleotides, {A, U, C, G}.

A weighted motif-similarity graph was therefore constructed and Louvain community detection method [56] in the igraph-python package [57] was applied for community detection among motif candidates. For each motif cluster, the candidate with the highest coverage (or most significant adjusted *p*-value if coverages are tied) was reported as the final motif pattern.

### Detection of co-occurring motifs

In case one motif may include two separate complementary regions, we designed a post-analysis to evaluate the co-occurrence of two discontinuous short motifs. The statistical significance of motif *X* and *Y* co-occur on *j* sequences can be calculated as follows [58]:

4 $$ {p}_j=\frac{\left(\begin{array}{c}N\\ {}j\end{array}\right)\left(\begin{array}{c}N-j\\ {}{N}_Y-j\end{array}\right)\left(\begin{array}{c}N-{N}_Y\\ {}{N}_X-j\end{array}\right)}{\left(\begin{array}{c}N\\ {}{N}_Y\end{array}\right)\left(\begin{array}{c}N\\ {}{N}_X\end{array}\right)} $$

5 $$ p- value=\sum \limits_{m\in \left\{j,j+1,\dots, N\right\}}{p}_m $$

where N denotes the total number of sequences in the input dataset; N_X_ and N_Y_ are the respective coverages of motif *X* and *Y*. In the Eq. 5, the *p*-value of two motifs co-occurring on *j* sequences is the sum of probabilities that two motifs co-occurring on more than *J* sequences. After the Bonferroni multiple test correction, when the adjusted *p*-value of two co-occurring motifs less than 0.05, it shows the observation of co-occurring motifs on *j* sequences is statistically significant. Meanwhile, for all possible co-occurring pairs, statistical significance was also assessed by adjusted *p*-values though random sampling. In addition, the Normalized Mutual Information (NMI) [59] was obtained to measure how frequently two motifs present at the same time.

6 $$ NMI=\frac{2\sum \limits_{x\in X}\sum \limits_{y\in Y}p\left(x,y\right)\log \left(\frac{p\left(x,y\right)}{p(x)p(y)}\right)}{-\sum \limits_{x\in X}p(x)\log \left(p(x)\right)-\sum \limits_{y\in Y}p(y)\log \left(p(y)\right)} $$

where *x* and* y* denote the appearance (0 or 1) of two motifs *X* and *Y*, respectively. In our study, a pair of motifs were considered as the possible co-occurring motifs if they had over 0.8 normalized mutual information and met both significance criteria.

### Cell culture, transfection, and RT- qPCR

An internal transfection experiment was performed to validate the predicted motif in SW620 cell. The protocol was as follows: 0.25 × 10^6^ human colorectal adenocarcinoma cell (SW620, American Type Culture Collection CCL-227) were grown in the Leibovitz’s L-15 medium supplemented with 10% FBS (Corning) and Penicillin-Streptomycin (ThermoFisher) for 24 h. Then the cells were washed Leibovitz’s L-15 medium supplemented with 10% Exosome-depleted FBS Media Supplement Heat Inactivated (SBI) and culture for total 48 h. 3  mL medium was collected every 24 h and stored at 4C°. At 48 h, the cells were washed with PBS and the miRNA was extracted by miRNeasy mini kit (Qiagen).

The exosomes were extracted using ExoQuick-TC (SBI). Cell culture medium with exosome-depleted FBS was collected for every 24 h. After removing the cell debris by centrifuge at 3000×g for 15 min, ExioQuick-TC were added into the medium with a 5:1 ratio and keep at 4C° for overnight. Next day, we centrifuged the mixture at 1500×g for 30 min at 4C° and re-suspended the pallet with 50 μL PBS with Protease Inhibitor Cocktail Set I (Millipore). mirVana mimic and customized mirVana mimic were purchased from Thermo Fisher Scientific. Then all mimics were transfected into SW620 with Lipofectamine RNAiMAX (Life Technologies).

Intracellular and exosomal total RNA (5 ng) was reverse transcribed with the Universal cDNA Synthesis Kit II (Exiqon) according to the manufacturer’s protocol. Diluted (1:40) cDNA samples were used for qPCR in a total volume of 10 μL using the ExiLENT SYBR® Green master mix and miRNA-specific primers (Exiqon). Relative amounts of intracellular and exosomal miRNAs were obtained using the 2^−ΔΔCt^ method. The UniSp6 RNA was used as the spike-in control and the small RNA U6 and SNORD44 were used for normalization of miRNA relative quantities in both cellular and exosomal preparations. RT-qPCR was performed with CFX Connect (Bio-Rad).

### Performance comparison

Using two exosomal miRNA datasets from Villarroya-Beltri et al. [12] and Santangelo et al. [13], a performance comparison was conducted on motif prediction among MDS^2^ and other methods, including MEME [15], DREME [43], COSMO [16], Improbizer [38], MERCI [60], DMINDA 2.0 [61] and MotifClick [47]. The experiment settings for each tool are shown in Table 2.

**Table 2:** Parameter settings for performance comparison among eight motif finding methods

Motif finding tools	Motif search size range	E-value or *p*-value threshold	Background model	Reference
MDS^2^	k ≥ 2	0.05	Large-scale sampling	
MEME	2 ≤ k ≤ 50	0.05	0-order Markov Model	[15]
DREME	–	0.05	K di-nucleotide shuffled input sequences	[43]
COSMO	2 ≤ k ≤ 8	–	0-order Markov Model	[16]
Improbizer	3 ≤ k ≤ 20	–	0-order Markov Model	[38]
MERCI	–	0.05	K di-nucleotide shuffled input sequences	[60]
DMINDA 2.0	k ≥ 6	0.05	–	[61]
MotifClick	k ≥ 4	–	–	[47]

## Results

**Fig. 3:**
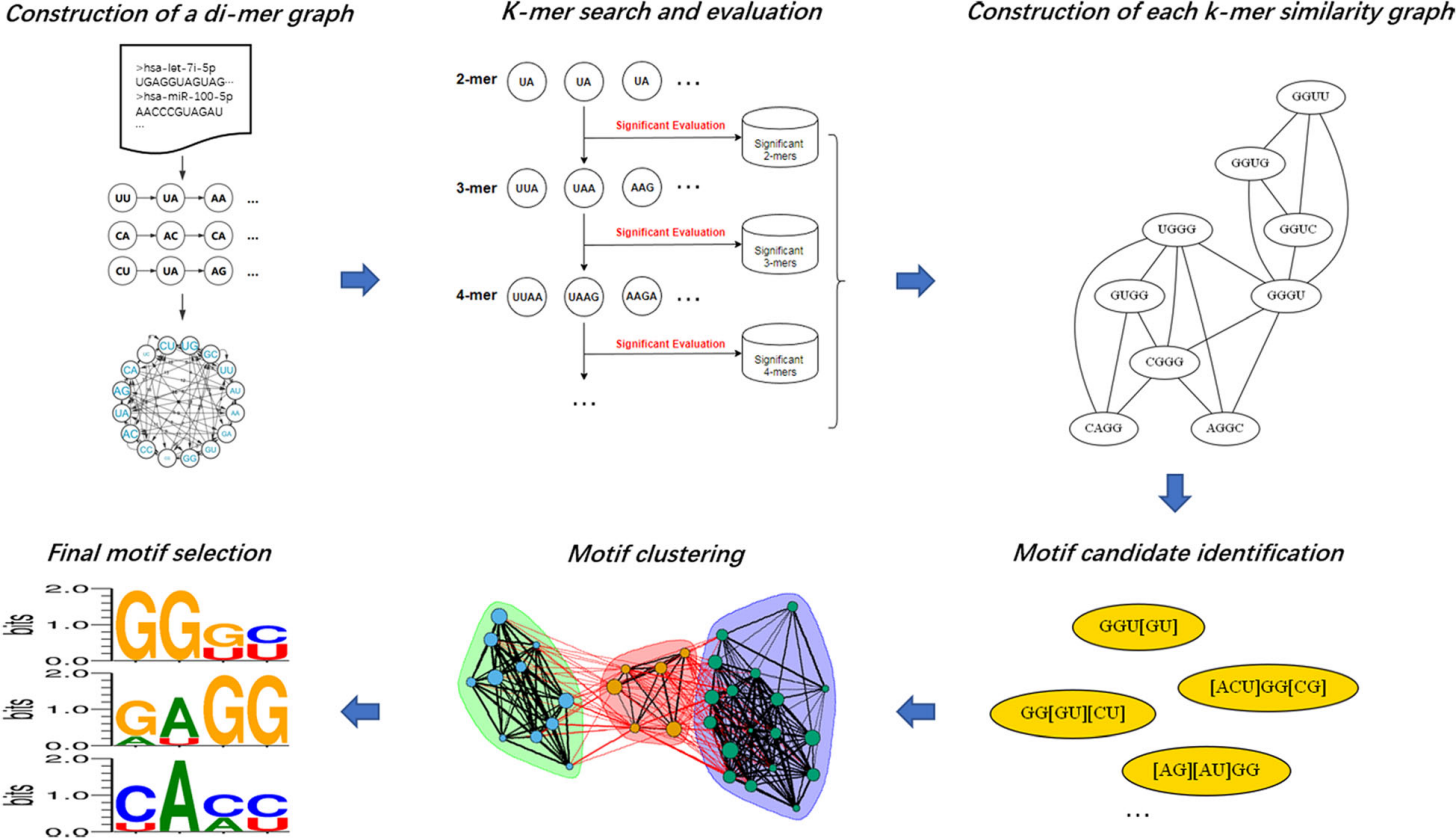
Schematic flowchart of proposed method on motif detection

The schematic flowchart in Fig. 3 showcases the computational procedure of the proposed method. This pipeline includes three major steps: 1) initialization that converts the input sequences to a di-mer graph; 2) motif candidate detection that firstly searches for significant *k*-mers on the initial di-mer graph and then identify possible motif candidates based the *k*-mer similarity graph; 3) motif summarization that performs graph clustering to find the most representative candidate as the final motif.

### Motifs responsible for loading exosomal miRNAs

To demonstrate the use and performance of the proposed method, we first re-analyzed the literature-reported exosomal miRNAs, e.g., 30 human miRNA sequences associated with loading protein hnRNPA2B1 in peripheral blood mononuclear cell [12] (Material and Methods). By using MDS^2^ seven motifs (3-5 bp long) were predicted (the full prediction result is shown in Figure S1, Additional file 1) where 3 motifs (two 3-mer motifs and one 4-mer motif) achieved the full coverage among given sequences. In another data including 103 mouse miRNA sequences from murine hepatocyte 3A cell [13], MDS^2^ identified 5 motifs (two 3-mer motifs and three 4-mer motifs are shown in Figure S2, Additional file 1) with the coverage from 70.9% to 98.1%.

**Fig. 4:**
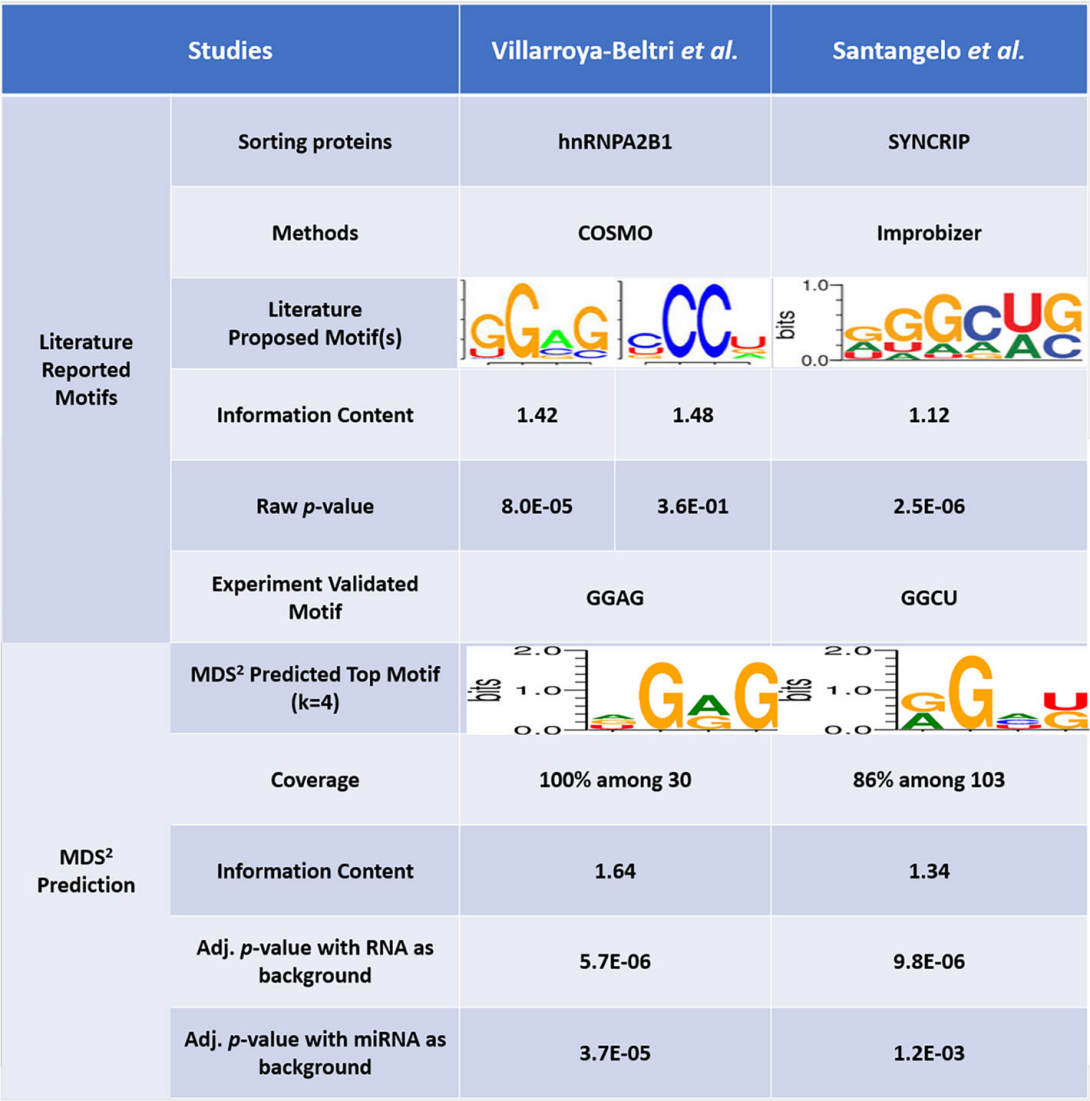
Prediction validation using reported motifs of exosomal miRNA in literature

First, we compared the predicted motifs by MDS^2^ with the experimental validated ones from the original studies and summarized the results in Fig. 4. Through multiple sequence alignment and motif finding using COSMO [16], Villarroya-Beltri et al. predicted two motifs associated with hnRNPA2B1 loading (Column 2) and successfully validated a 4-mer, GGAG, on regulating the expressions of two test miRNAs (miR-17 and miR-601) in exosomes. Using the same data, MDS^2^ predicted three motifs (shown in Figure S1, Additional file 1), among which the top-ranked motif [AGU]G[AG]G (adjusted *p*-values: 5.6E-06 (RNA) and 3.7E-05 (miRNA)) covers the above validated GGAG (adjusted *p*-value: 5.8E-07 (RNA) and 5.4E-06 (miRNA)). This top-ranked motif is not only appearing in all input exosomal miRNA sequences, but also has a high IC that 50% of positions are conserved. Moreover, MDS^2^ also successfully identified a 3-mer motif [AUG]G[GA] (adjusted *p*-value: 1.3E-04 (RNA) and 2.6E-04 (miRNA)) that contains another RNA-binding motif of hnRNPA2B1, AGG (adjusted *p*-value: 4.5E-06 (RNA) and 1.0E-05 (miRNA)), that is recently reported through a study of the crystal structure of protein-RNA complex [62]. In the case of murine hepatocyte 3A cell (Column 3 in Fig. 4), Santangelo et al. found that a 4-mer motif, GGCU, is responsible for loading intercellular miRNAs into exosome [13] through motif analysis using Improbizer [38]. Similar to the pervious results, the top-rank prediction from MDS^2^ [AG]G[ACU][UG] (adjusted *p*-value: 9.8E-06 (RNA) and 1.2E-03 (miRNA)), covers the validated motif GGCU (adjusted *p*-value: 7.9E-06 (RNA) and 5.9E-04 (miRNA)) (Fig. 4).

### Performance comparison with other motif finding methods on exosomal miRNAs

**Fig. 5:**
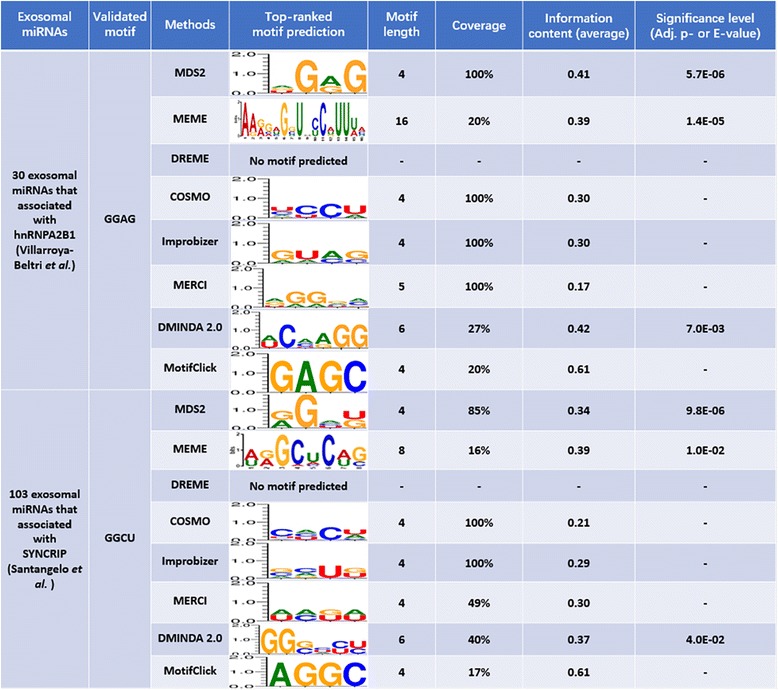
Performance comparisons on exosomal miRNA motif prediction

Using these two validated datasets, we conducted a performance comparison on motif prediction with other methods. The parameter setting for each method shown in Table 2 and the prediction results are shown in the Fig. 5 (only includes the top-ranked predicted motif of each tool).

It is highly encouraging that MDS^2^ can successfully identify real exosomal motifs and report them as the top-ranked prediction (Fig. 5). Moreover, predicted motifs from MDS^2^ usually possess higher coverage and IC than other tools. In contrast, other existing tools tend to predict motifs that are more general and have more complex composition. For example, the COSMO-predicted motifs had no conserved position and produced low IC. Although some motifs from COSMO, Improbizer and MERCI can cover 100% of input sequences, the patterns contain too many variations that failed to provide any useful information for the further experiment validation. In addition, after conducting the same analysis (search motif from 3 bp to 50 bp) using MEME [15], we found MEME predicted a 10-bp and 8-bp long motifs for two datasets (Fig. 5), which are impractically long for the exosomal miRNA. In both datasets, DREME failed to detect any motifs. The graph-based approach, MotifClick, predicted two 4-mers as the final motifs, which obviously lack representativeness since only about 20% of input sequences contain the predicted motifs.

Overall, we demonstrated that MDS^2^ is efficient to detect motifs with a balanced coverage and explicitness, suitable for the motif detection among short sequences. Especially, MDS^2^ provides very reliable motif prediction when the negative information is limited, which benefits from the unbiased *k*-mer evaluation based on the large-scale sampling.

### Assessment of motif detection sensitivity in different input sets

It is notable that exosomal content reported by different studies could be heterogeneous, even on the same cell type, which is partially caused by the technical variations across different detection methodologies. Here we assess the robustness of MDS^2^ using two different datasets on human colon cancer cell (SW620). By re-analyzed the expression profiles of exosomal miRNAs in SW620 [30] (GEO accession number: GSE21350), we detect 112 miRNAs that have consistent expressions in all cell samples and exosome samples, respectively, (Table S1, Additional file 1) in contrast to the 39 exosomal miRNAs reported in EVpedia database.

**Fig. 6:**
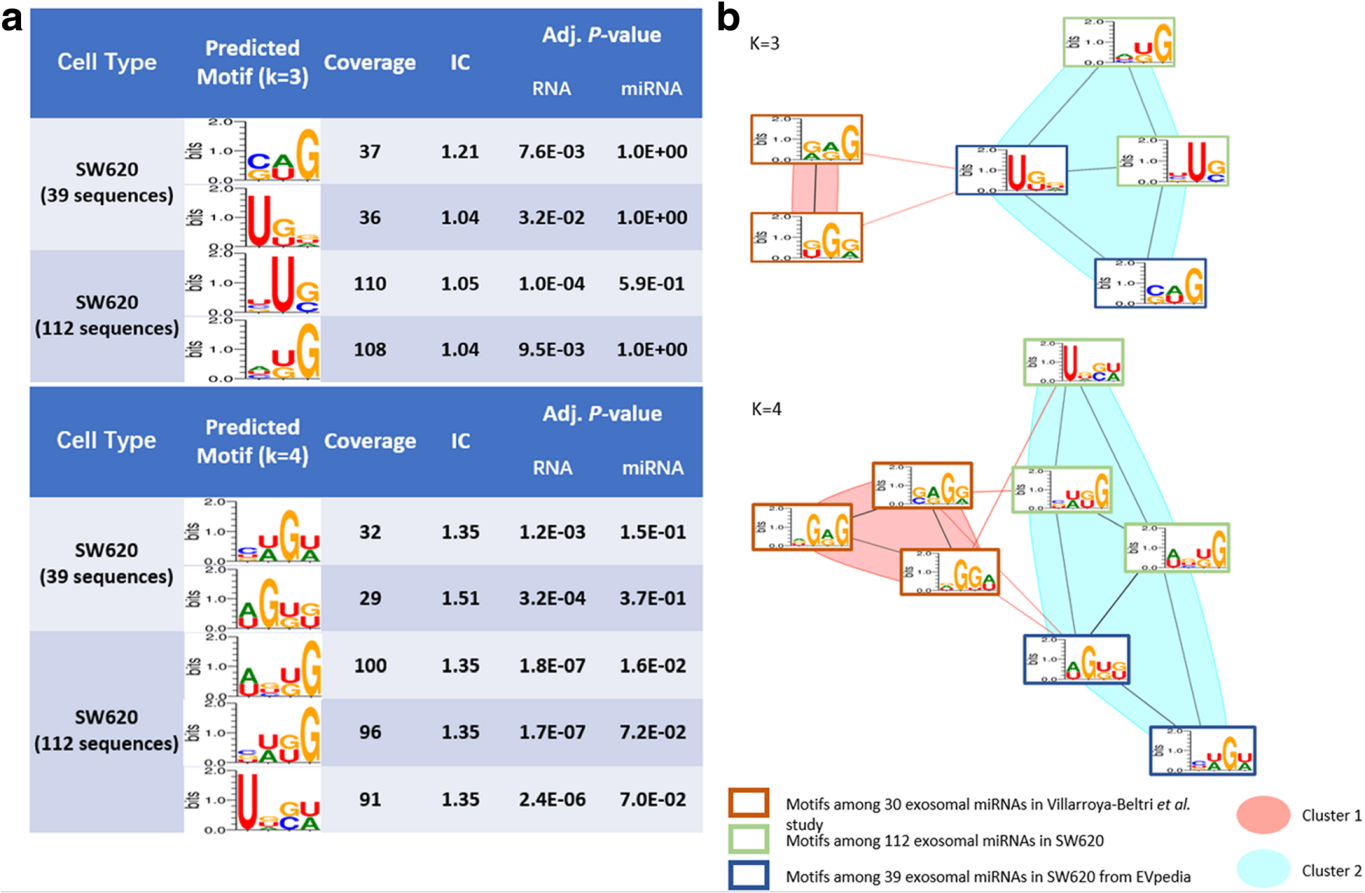
Predicted motifs of exosomal miRNA in SW620 cell using MDS^2^. **a** The predicted motifs from two datasets (K = 3, 4). **b** Motif clustering among the predicted motifs from two datasets (K = 3, 4)

MDS^2^ predicted a total of eight motifs on two datasets (Figures S3 and S4, Additional file 1). To assess the consistency between two predictions, we mixed all predicted motifs, calculated the similarity, and applied motif clustering. We found those motifs fell into two clusters (Fig. 6b). As expected, the motifs from two SW620 datasets were congregated into the same group, which is distinct from the motifs reported in Villarroya-Beltri et al. study. This observation indicates the predicted motifs from two datasets under SW620 are highly similar.

**Fig. 7:**
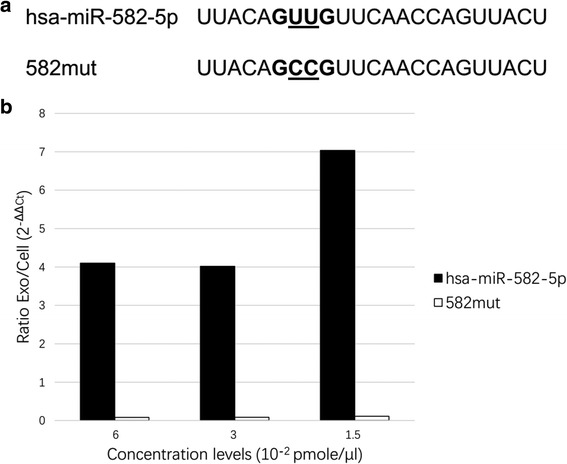
Experimental validation on the predicted motif “GUUG” in SW620 cell line. **a** Sequences of wild-type exosomal miRNA hsa-miR-582-5p and its mutated version, 582mut, that does not contain the motif (**b**) Intercellular and exosomal levels of hsa-miR-582-5p and 582mut using RT-qPCR based on three plasmid transfection concentrations

Moreover, to test if MDS^2^-predicted motifs are truly responsible for exosomal miRNA loading in SW620 cell, we focused on the significant 4-mer motif, [CGU][UA][GU]G, (coverage: 86%, adjusted *p*-values: 1.7E-07 (RNA) and 7.2E-02 (miRNA)) from our top-ranked prediction to validate experimentally. To ensure a successful validation, we decided to choose a miRNA that has detectable expressions in both SW620 cell and its exosomes so that we can assess the motif effect on exosomal miRNA loading by examining the abundance change through transfection analysis. MiR-582-5p is therefore selected and the associated 4-mer in the sequence is GUUG. Note that GUUC alone has a coverage of 14 out of 112 in the original dataset (adjusted *p*-values: 7.2E-03 (RNA) and 2.4E-02 (miRNA)). Both wildtype sequence and mutated sequence (without GUUG) were transfected into SW620 cell at three difference levels of concentration: 6, 3 and 1.5 (10^− 2^ pmole/μL). The cellular and exosomal expressions of both sequences were assessed using RT-qPCR. In Fig. 7b, it is clear that across three concentration levels, the Exo/Cell ratio of miR-582-5p is significantly higher than the mutated sequences 582mut, which indicates that the motif GUUG are associated with loading more miRNAs into exosomes.

### Motifs associated with longer RNAs

In addition to the short miRNAs, we utilized the following case studies to demonstrate the performance of MDS^2^ on longer input sequence.

#### Test example 1: Motifs on exosomal mRNAs

**Fig. 8:**
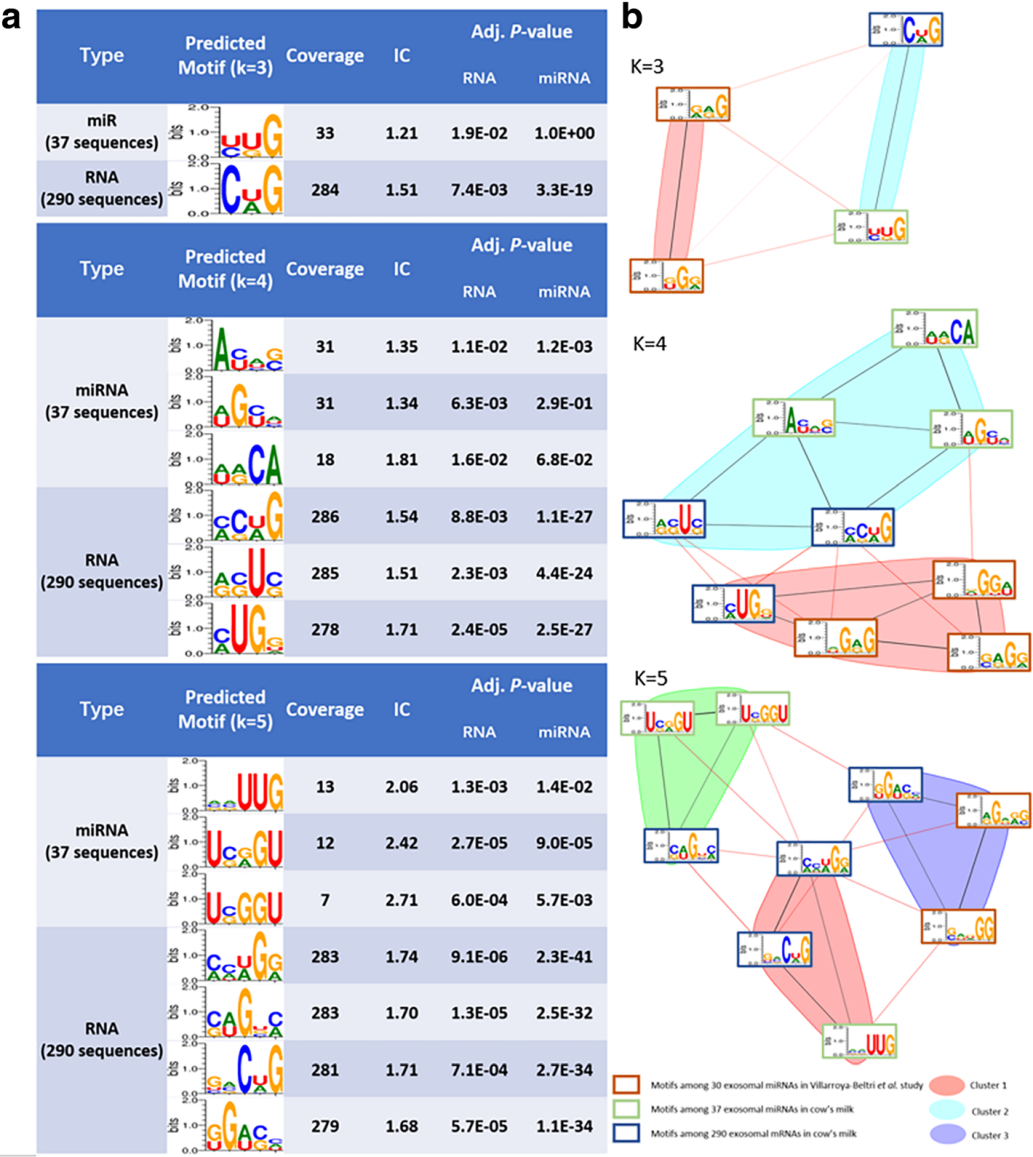
Exosomal miRNAs and mRNAs share similar motifs in bovine milk’s exosomes. **a** Motif prediction on exosomal miRNAs and mRNAs (K = 3, 4, 5); **b** Motif clustering among the predicted motifs from exosomal miRNAs and mRNAs datasets (K = 3, 4, 5)

Based on an in-house data of 290 bovine-milk exosomal mRNAs, which includes mostly fragments with length range of 50~ 171 bps, MDS^2^ detected 22 motifs of different lengths (3-7 bps, full list shown in Figure S5, Additional file 1). Figure 8a shows the top predictions of 3-5 bps. For example, the most promising 4-mer motif, [CA]UG[GUA] (adjusted *p*-value in RNA background: 2.4E-05), has a 96% coverage (278 out of 290) among the input sequences and the best 5-mer motif, [CA][CAU][UA]G[GA] (adjusted *p*-value in RNA background: 9.1E-06), covers 283 out 290 sequences.

Of particular interest is that, when compared the mRNA motifs with those predicted from 37 bovine-exosome miRNAs in the same analysis (listed in Figure S6, Additional file 1), we found miRNA motifs are mostly 3-5 bp long while mRNA motifs can be up to 7 bps long. Similar to the assessment for the exosomal miRNA motifs in SW620 cell, we mixed the predicted motifs from Villarroya-Beltri et al. study with the predicted motifs in cow’s milk exosomal RNAs. As shown in Fig.8b, at the size of 3, 4 and 5, respectively, the motifs of the exosomal RNAs in cow’s milk were highly merged into the same clusters while the motifs from Villarroya-Beltri et al. study always form their own cluster. This indicates a high level of similarity among those motifs from cow’s milk exosomal RNAs and also implies that short miRNAs and mRNA fragments may share the common sorting mechanism into exosome, regardless of the length or type of the RNA.

#### Test example 2: Motifs on miRNA-mRNA binding sites

Similarly, MDS^2^ can detect the regulatory motif of a miRNA on their mRNA targets. For illustration, we used 1064 binding sites of hsa-miR-92a-3p, a miRNA with the most targets in CLASH data [8]. Based on the different base-pairing patterns, those binding sites were categorized into 5 different classes according to the folding class definition proposed in [8].

**Fig. 9:**
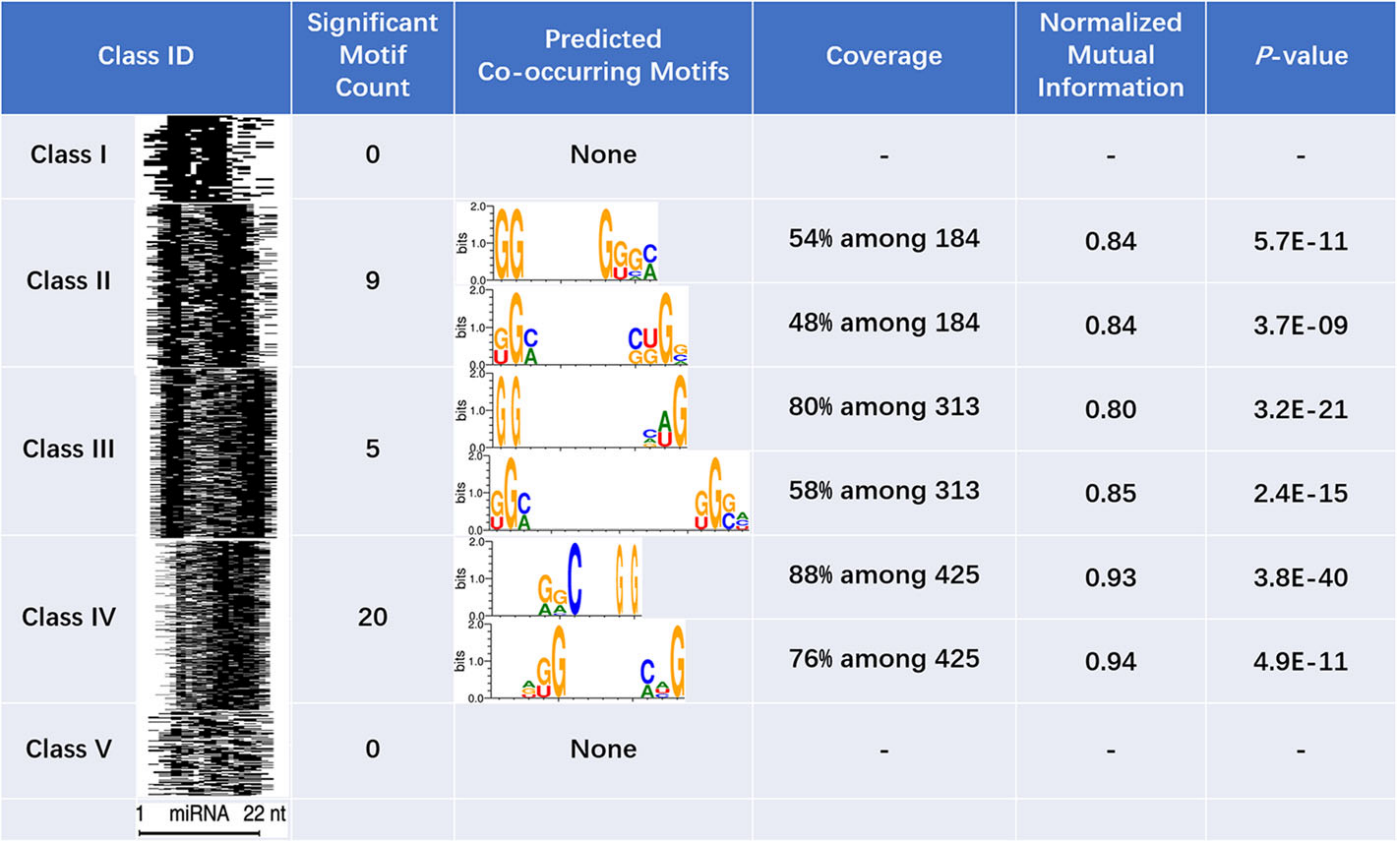
Detection of joint motifs among the targets of hsa-miR-92a-3p. 1064 interaction sites were categorized into five binding classes with different binding patterns (A grayscale heatmap presented in column 1 to show the location of the motifs on the sequences.)

It is well-studied that human miRNA-mRNA interaction does not require consecutive base-pairing at their binding sites (Separate biding regions shown in Fig. 9). As described in the Method, a post-analysis was integrated in MDS^2^ to evaluate the pair-wise co-occurrence among motifs and to identify joint motifs. Here, we performed joint motif detection on each class.

Figure 9 shows that the identified motifs are highly consistent with interaction pattern in each class. For instance, the distance range between sub-motifs are 3–6 bps in class II and 3-10 bps in class III, respectively. On the contrary, classes I and V don’t have significant joint motifs, as reflected in their patterns. Moreover, the high-level of mutual information of the predicted motifs in classes II-IV also suggested the significant association between two sub-motifs. In general, our observation is concordant with the binding patterns stated in [8], e.g., class III involves base-pairing outside of seed region while the fairly-distributed pairing was dominating in class V.

#### Test example 3: Motif of AGO-binding to mRNAs

Erhard’s AGO2-PAR-CLIP data was used, which includes 12-thousand AGO2-binding sites on human transcripts in two B cell lines: BCBL1 and DG75 [52]. We chose 545 sequences of 23 ~ 114 bps long that were consistently and highly expressed across all six samples.

**Fig. 10:**
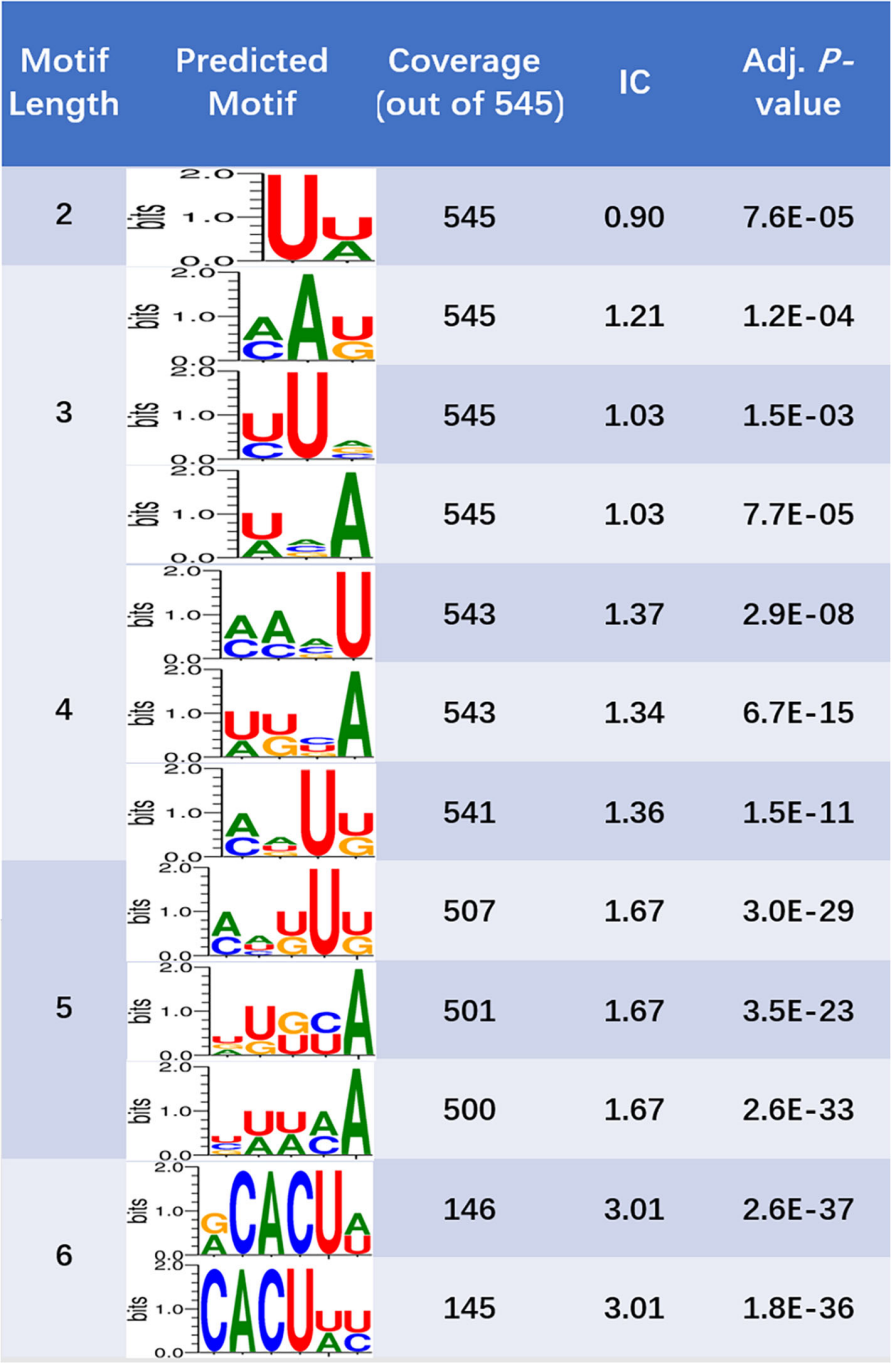
Predicted motifs of AGO2 protein binding sites in human

Using MDS^2^ 12 motifs of 2-5 bps were reported, with 27% to 100% coverage (Fig. 10). As a well-studied RNA binding protein in human, AGO2 has five known RNA binding regions that are different in length (2-43 bps). The predictions show that there are significant motifs associated with each of those regions. In contrast, motifs predicted from MEME was 18 bps long and only cover ~ 30% of the input sequences while the top-ranked 6-mer predicted by DREME only covers ~ 18% of input sequences.

### The MDS^2^ webserver

**Fig. 11:**
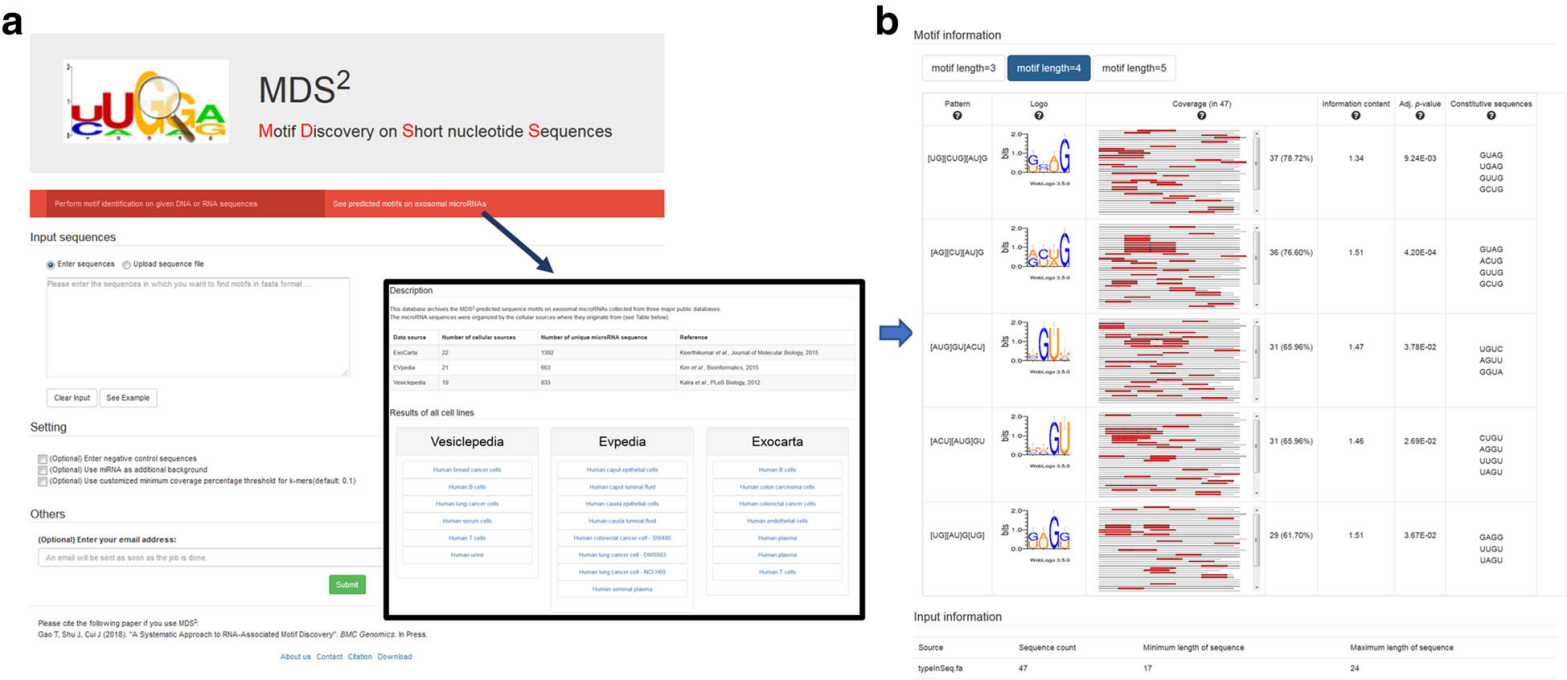
Illustration of MDS^2^ web-server. **a** A submission page for user sequence input and a database interface to access cell-specific motifs predicted on a variety of extracellular miRNAs; **b** an example result page that visualizes predicted motifs along with statistics

The MDS^2^ web-server was developed based on the proposed methods and is currently available at http://sbbi-panda.unl.edu/MDS2/. A standalone package of MDS^2^ is also available for download at the website. In addition, we have compiled an online database that included all cell-specific predicted motifs for a variety of extracellular miRNAs (Fig. 11).

## Discussion

Recent advances in high-throughput sequencing technologies have enabled the mechanistic study of RNA processing, sorting, and regulation through screening for RNA-protein and RNA-RNA interaction sites. In this study, we have clearly demonstrated that motif analysis on short exosomal miRNAs can be fundamentally important to understand the mechanisms of miRNA loading and intercellular transfer. However, revisit of motif finding analysis in these new research topics inevitably encountered new challenges. For example, existing motif finding approaches that need well-defined negative datasets to evaluate the statistical significance of overrepresented sequences often fail to detect significant short motifs, according to the literature [12, 13] and our own test. In the MEME method, the “significance” of a potential motif is decided by E-value, which is “an estimate of the number of (equally or more interesting) motifs one would expect to find by chance if the letters in the input sequences were shuffled” [15]. However, the shuffled input sequence may not be able to represent an unbiased background, especially when the input sequences are few and short, like the exosomal miRNA cases in this study. Likewise, the background is extremely critical to DREME since it uses the exact fisher test to define a “significant” motif. This process can be very misleading without the high quality negative dataset.

On the other hand, although very little is known about the binding site between miRNAs and transporter proteins, many studies have attempted resolve the structure of AGO-miRNA to explore the miRNA-binding regions on AGO proteins [63–66]. AGO protein contains two functional domains that may interact with miRNA: N-PAZ domain that usually plays the 3′-nucleotide-binding on miRNAs (around 3 bp long) [46] and MID-PIWI domain that interact with 11th or 15th nucleotide on miRNAs [63]. The evidences showed that the functional AGO-binding region on miRNA could be very short, even shorter than the seed region (position 2–8 on 3′ side of miRNA). Moreover, a recent study reported that hnRNPA2/B1, the exosomal miRNA sorting protein, contains two short RNA recognition motifs (AGG and UAG) in the crystal structure of protein-RNA complex [62]. These evidences may indicate that the functional motifs on miRNA sequences can be as short as 3-4 bp long.

Note that the design of large-scale sampling introduces extra computational time which might be the major bottleneck of this pipeline, although the 2-mer graph design searching for longer *k*-mer candidate is more time efficient compared to the enumeration of all *k*-mers (Fig. S7, Additional file 1). Using our webserver, a job of 184 sequence with average length of 65 bps needs two hours to complete and the significant evaluation of potential motifs takes about 64.5% of total running time. To overcome this computational drawback, we have implemented the optional parameters that allow user to apply more aggressive filtering standard to reduce the number of redundant *k*-mers and motif candidates and reduce the computing cost. While the MDS^2^ webserver is constrained for moderate-size tasks (less than 12,000 nucleotides per submission), the standalone package is available for user to use the tool locally. As demonstrated in this study, it is highly promising and encouraging to perform systematic motif analysis using MDS^2^ to understand the novel biology in extracellular RNA sorting and trafficking, as well as in other similar RNA and single-stranded DNA applications.

## Conclusions

In this study, we proposed a new method, MDS^2^ specifically to tackle the newly-identified challenges in motif finding on short nucleotide sequences. MDS^2^ initializes the motif search from overrepresented di-mers among given sequences and then expends into longer motifs through significant path finding in graphs. More importantly, MDS^2^ has demonstrated competitive performance in finding motifs with exceptional high coverage and explicitness. The predictions were validated through literature search and internal experimental test. Instead of gauging the over-representation among input sequences from a possibly limited collection, it integrates a very large-scale random sampling to evaluate statistical significance of all motif candidates and avoid the construction of a questionable negative dataset. Moreover, it is notable that MDS^2^ is a parameter-free algorithm that requires only the input sequences. We expect the method developed in this study can provide the community a promising solution on motif discovery on short nucleotide sequences.

## Additional file

Additional file 1: Supplementary Figures and Tables. (PDF 1044 kb)

## Abbreviations

AGO: Argonaute
ChIP-Seq: Chromatin Immunoprecipitation Sequencing
CLASH: The crosslinking, ligation, and sequencing of hybrids
EM: Expectation–Maximization
MRA: Motif Recognition Algorithm
NMI: Normalized Mutual Information
PAR-CLIP: Photoactivatable Ribonucleoside-Enhanced Crosslinking and Immunoprecipitation
PCC: Pearson Correlation Coefficient
PWM: Position Weight Matrix
RIP-Seq: RNA Immunoprecipitation Sequencing
TF: Transcription Factor

## References

[1] D’haeseleer P (2006). How does DNA sequence motif discovery work?. Nat Biotechnol [Internet].

[2] Bailey TL, Elkan C. Fitting a mixture model by expectation maximization to discover motifs in Bipolymers. Proc Second Int Conf Intell Syst Mol Biol [Internet]. 1994;28–36. Available from: http://citeseerx.ist.psu.edu/viewdoc/download?doi=10.1.1.121.7056&rep=rep1&type=pdf%5Cnhttp://www.cs.utoronto.ca/~brudno/csc2417_10/10.1.1.121.7056.pdf 7584402

[3] Park PJ (2009). ChIP–seq: advantages and challenges of a maturing technology. Nat Rev Genet [Internet].

[4] Hafner M, Lianoglou S, Tuschl T, Betel D (2012). Genome-wide identification of miRNA targets by PAR-CLIP. Methods [Internet].

[5] Hafner M, Landthaler M, Burger L, Khorshid M, Hausser J, Berninger P, et al. Transcriptome-wide identification of RNA-binding protein and MicroRNA target sites by PAR-CLIP. Cell. 2010;141:129–41. 10.1016/j.cell.2010.03.009 PMC2861495 20371350

[6] Erhard F, Dölken L, Jaskiewicz L, Zimmer R (2013). PARma: identification of microRNA target sites in AGO-PAR-CLIP data. Genome Biol [Internet].

[7] Kudla G, Granneman S, Hahn D, Beggs JD, Tollervey D (2011). Cross-linking, ligation, and sequencing of hybrids reveals RNA-RNA interactions in yeast. Proc Natl Acad Sci [Internet].

[8] Helwak A, Kudla G, Dudnakova T, Tollervey D. Mapping the human miRNA interactome by CLASH reveals frequent noncanonical binding. Cell [Internet]. 2013;153:654–65. Available from: http://www.ncbi.nlm.nih.gov/pubmed/23622248 10.1016/j.cell.2013.03.043 PMC3650559 23622248

[9] Kheradpour P, Kellis M (2014). Systematic discovery and characterization of regulatory motifs in ENCODE TF binding experiments. Nucleic Acids Res.

[10] Khan A, Fornes O, Stigliani A, Gheorghe M, Castro-Mondragon JA, van der Lee R, et al. JASPAR 2018: update of the open-access database of transcription factor binding profiles and its web framework. Nucleic Acids Res [Internet]. 2017; Available from: http://academic.oup.com/nar/article/doi/10.1093/nar/gkx1126/4621338 10.1093/nar/gkx1126 PMC5753243 29140473

[11] Zealy RW, Wrenn SP, Davila S, Min K-W, Yoon J-H. microRNA-binding proteins: specificity and function. Wiley Interdiscip Rev RNA [Internet] John Wiley & Sons, Inc; 2017 [cited 2017 Jul 30];e1414. Available from: http://doi.wiley.com/10.1002/wrna.1414 10.1002/wrna.1414 28130820

[12] Villarroya-Beltri C, Gutiérrez-Vázquez C, Sánchez-Cabo F, Pérez-Hernández D, Vázquez J, Martin-Cofreces N, et al. Sumoylated hnRNPA2B1 controls the sorting of miRNAs into exosomes through binding to specific motifs. Nat Commun [Internet] Nature Publishing Group; 2013 [cited 2017 May 2];4:2980. Available from: http://www.nature.com/doifinder/10.1038/ncomms3980 10.1038/ncomms3980 PMC3905700 24356509

[13] Santangelo L, Giurato G, Cicchini C, Montaldo C, Mancone C, Tarallo R (2016). The RNA-binding protein SYNCRIP is a component of the hepatocyte Exosomal machinery controlling MicroRNA sorting. Cell Rep.

[14] Moore MJ, Scheel TKH, Luna JM, Park CY, Fak JJ, Nishiuchi E (2015). miRNA-target chimeras reveal miRNA 3′-end pairing as a major determinant of Argonaute target specificity. Nat. Commun. [Internet].

[15] Bailey TL, Boden M, Buske FA, Frith M, Grant CE, Clementi L, et al. MEME suite: tools for motif discovery and searching. Nucleic Acids Res. 2009;37 10.1093/nar/gkp335 PMC2703892 19458158

[16] Bembom O, Keles S, van der Laan MJ. Supervised detection of conserved motifs in DNA sequences with Cosmo. Stat Appl Genet Mol Biol [Internet]. 2007:6. Available from: http://www.degruyter.com/view/j/sagmb.2007.6.issue-1/sagmb.2007.6.1.1260/sagmb.2007.6.1.1260.xml 10.2202/1544-6115.1260 17402923

[17] Valadi H, Ekström K, Bossios A, Sjöstrand M, Lee JJ, Lötvall JO (2007). Exosome-mediated transfer of mRNAs and microRNAs is a novel mechanism of genetic exchange between cells. Nat Cell Biol [Internet].

[18] Mathivanan S, Ji H, Simpson RJJ. Exosomes: extracellular organelles important in intercellular communication. J Proteome. 2010:1907–20. 10.1016/j.jprot.2010.06.006 20601276

[19] Simons M, Raposo G (2009). Exosomes--vesicular carriers for intercellular communication. Curr Opin Cell Biol.

[20] Crescitelli R, Lässer C, Szabó TGG, Kittel A, Eldh M, Dianzani I (2013). Distinct RNA profiles in subpopulations of extracellular vesicles: apoptotic bodies, microvesicles and exosomes. J Extracell Vesicles [Internet].

[21] Liang X, Zhang L, Wang S, Han Q, Zhao RC (2016). Exosomes secreted by mesenchymal stem cells promote endothelial cell angiogenesis by transferring miR-125a. J Cell Sci [Internet].

[22] Gong M, Yu B, Wang J, Wang Y, Liu M, Paul C, et al. Mesenchymal stem cells release exosomes that transfer miRNAs to endothelial cells and promote angiogenesis. Oncotarget [Internet] 2017;8:45200–45212. Available from: http://www.ncbi.nlm.nih.gov/pubmed/28423355%0Ahttp://www.pubmedcentral.nih.gov/articlerender.fcgi?artid=PMC5542178%0Ahttp://www.oncotarget.com/fulltext/16778 10.18632/oncotarget.16778 PMC5542178 28423355

[23] Ferrante SCC, Nadler EPP, Pillai DKK, Hubal MJJ, Wang Z, Wang JMM (2015). Adipocyte-derived exosomal miRNAs: a novel mechanism for obesity-related disease. Pediatr Res [Internet].

[24] Chen Y, Buyel JJJ, MJWJW H, Siegel F, Pan R, Naumann J (2016). Exosomal microRNA miR-92a concentration in serum reflects human brown fat activity. Nat Commun [Internet].

[25] Munagala R, Aqil F, RCC G (2016). Exosomal miRNAs as biomarkers of recurrent lung cancer. Tumor Biol.

[26] Thind A, Wilson C. Exosomal miRNAs as cancer biomarkers and therapeutic targets. J Extracell Vesicles. 2016; 10.3402/jev.v5.31292 PMC4954869 27440105

[27] Ogata-Kawata H, Izumiya M, Kurioka D, Honma Y, Yamada Y, Furuta K, et al. Circulating exosomal microRNAs as biomarkers of colon cancer. PLoS One. 2014;9 10.1371/journal.pone.0092921 PMC3976275 24705249

[28] Hessvik NPP, Sandvig K, Llorente A. Exosomal miRNAs as biomarkers for prostate cancer. Front Genet. 2013; 10.3389/fgene.2013.00036 PMC3604630 23519132

[29] Zhang J, Li S, Li L, Li M, Guo C, Yao J, et al. Exosome and Exosomal MicroRNA: trafficking, sorting, and function. Genomics Proteomics Bioinformatics [Internet]. 2015 [cited 2017 May 2];13:17–24. Available from: http://www.sciencedirect.com/science/article/pii/S167202291500011X 10.1016/j.gpb.2015.02.001 PMC4411500 25724326

[30] Ohshima K, Inoue K, Fujiwara A, Hatakeyama K, Kanto K, Watanabe Y, et al. Let-7 microRNA family is selectively secreted into the extracellular environment via exosomes in a metastatic gastric cancer cell line. PLoS One. 2010;5 10.1371/journal.pone.0013247 PMC2951912 20949044

[31] Dreux M, Garaigorta U, Boyd B, Décembre E, Chung J, Whitten-Bauer C (2012). Short-range exosomal transfer of viral RNA from infected cells to plasmacytoid dendritic cells triggers innate immunity. Cell Host Microbe.

[32] Singh PP, Li L, Schorey JS (2015). Exosomal RNA from mycobacterium tuberculosis-infected cells is functional in recipient macrophages. Traffic.

[33] Huang X, Yuan T, Tschannen M, Sun Z, Jacob H, Du M, et al. Characterization of human plasma-derived exosomal RNAs by deep sequencing. BMC Genomics [Internet]. 2013;14:–319. Available from: http://bmcgenomics.biomedcentral.com/articles/10.1186/1471-2164-14-319 10.1186/1471-2164-14-319 PMC3653748 23663360

[34] Ekström K. Exosomal shuttle RNA: Universoty Gothenbg; 2008.

[35] Lässer C (2012). Exosomal RNA as biomarkers and the therapeutic potential of exosome vectors. Expert Opin Biol Ther [Internet].

[36] Eldh M, Ekström K, Valadi H, Sjöstrand M, Olsson B, Jernås M (2010). Exosomes communicate protective messages during oxidative stress; possible role of Exosomal shuttle RNA. PLoS One.

[37] Cha DJ, Franklin JL, Dou Y, Liu Q, Higginbotham JN, Beckler MD, et al. KRAS-dependent sorting of miRNA to exosomes. Elife [Internet]. 2015 [cited 2017 May 2];4. Available from: http://elifesciences.org/lookup/doi/10.7554/eLife.07197 10.7554/eLife.07197 PMC4510696 26132860

[38] Ao W, Gaudet J, Kent WJ, Muttumu S, Mango SE. Environmentally induced foregut remodeling by PHA-4/FoxA and DAF-12/NHR. Science (80- ) [Internet]. 2004 [cited 2017 May 27];305:1743–1746. Available from: http://www.ncbi.nlm.nih.gov/pubmed/15375261 10.1126/science.1102216 15375261

[39] Zhao Y, Stormo GD. Quantitative analysis demonstrates most transcription factors require only simple models of specificity. Nat Biotechnol. 2011:480–3. 10.1038/nbt.1893 PMC3111930 21654662

[40] Orenstein Y, Mick E, Shamir R. RAP: accurate and fast motif finding based on protein-binding microarray data. J Comput Biol [Internet]. 2013;20:375–382. Available from: http://eutils.ncbi.nlm.nih.gov/entrez/eutils/elink.fcgi?dbfrom=pubmed&id=23464877&retmode=ref&cmd=prlinks%5Cnpapers2://publication/doi/10.1089/cmb.2012.0253%5Cnfile:///Users/Calixto/Papers2/Articles/2013/Orenstein/J Comput Biol/J Comput Biol 2013 Orenstei. 10.1089/cmb.2012.0253 PMC3646338 23464877

[41] Jolma A, Kivioja T, Toivonen J, Cheng L, Wei G, Enge M (2010). Multiplexed massively parallel SELEX for characterization of human transcription factor binding specificities. Genome Res.

[42] Nitta KR, Jolma A, Yin Y, Morgunova E, Kivioja T, Akhtar J, et al. Conservation of transcription factor binding specificities across 600 million years of bilateria evolution. elife. 2015;2015 10.7554/eLife.04837 PMC4362205 25779349

[43] Bailey TLL (2011). DREME: motif discovery in transcription factor ChIP-seq data. Bioinformatics.

[44] Willms E, Johansson HJ, Mäger I, Lee Y, Blomberg KEM, Sadik M (2016). Cells release subpopulations of exosomes with distinct molecular and biological properties. Sci Rep [Internet].

[45] Ko J, Carpenter E, Issadore D (2016). Detection and isolation of circulating exosomes and microvesicles for cancer monitoring and diagnostics using micro−/nano-based devices. Analyst [Internet].

[46] Fratkin E, Naughton BT, Brutlag DL, Batzoglou S. MotifCut: regulatory motifs finding with maximum density subgraphs. Bioinformatics [Internet]. 2006 [cited 2017 Dec 22];22:e150–e157. Available from: http://www.ncbi.nlm.nih.gov/pubmed/16873465 10.1093/bioinformatics/btl243 16873465

[47] Zhang S, Li S, Niu M, Pham PT, Su Z. MotifClick: prediction of cis-regulatory binding sites via merging cliques. BMC Bioinformatics [Internet]. 2011 [cited 2017 Dec 22];12:238. Available from: http://www.ncbi.nlm.nih.gov/pubmed/21679436 10.1186/1471-2105-12-238 PMC3225181 21679436

[48] Keerthikumar S, Chisanga D, Ariyaratne D, Al Saffar H, Anand S, Zhao K (2016). ExoCarta: a web-based compendium of Exosomal cargo. J Mol Biol.

[49] Kim DK, Lee J, Kim SRHSRH, Choi DS, Yoon YJ, Kim JH, et al. EVpedia: a community web portal for extracellular vesicles research. Bioinformatics [Internet]. 2015;31:933–939. Available from: http://www.ncbi.nlm.nih.gov/pubmed/25388151 10.1093/bioinformatics/btu741 PMC4375401 25388151

[50] Kalra H, Simpson RJ, Ji H, Aikawa E, Altevogt P, Askenase P, et al. Vesiclepedia: a compendium for extracellular vesicles with continuous community annotation. PLoS Biol. 2012;10 10.1371/journal.pbio.1001450 PMC3525526 23271954

[51] Helwak A, Kudla G, Dudnakova T, Tollervey D. Mapping the human miRNA interactome by CLASH reveals frequent noncanonical binding. Cell [Internet]. 2013;153:654–665. Available from: http://www.ncbi.nlm.nih.gov/pubmed/23622248. 10.1016/j.cell.2013.03.043 PMC3650559 23622248

[52] Erhard F, Haas J, Lieber D, Malterer G, Jaskiewicz L, Zavolan M, et al. Widespread context dependency of microRNA-mediated regulation. Genome Res [Internet] Cold Spring Harbor Laboratory Press; 2014 [cited 2017 Jul 8];24:906–919. Available from: http://www.ncbi.nlm.nih.gov/pubmed/24668909. 10.1101/gr.166702.113 PMC4032855 24668909

[53] Kullback S, Leibler RA. On information and sufficiency. Ann Math Stat [Internet] Institute of Mathematical Statistics; 1951 [cited 2018 Jan 24];22:79–86. Available from: http://projecteuclid.org/euclid.aoms/1177729694

[54] Tanaka E, Bailey T, Grant CE, Noble WS, Keich U. Improved similarity scores for comparing motifs. Bioinformatics [Internet] Oxford University Press; 2011 [cited 2017 Dec 7];27:1603–1609. Available from: http://www.ncbi.nlm.nih.gov/pubmed/21543443 10.1093/bioinformatics/btr257 PMC3106196 21543443

[55] Pietrokovski S (1996). Searching databases of conserved sequence regions by aligning protein multiple-alignments. Nucleic Acids Res.

[56] Blondel VDD, Guillaume J-L, Lambiotte R, Lefebvre E. Fast unfolding of community hierarchies in large networks. J Stat Mech Theory Exp [Internet]. 2008:1–6. Available from: http://iopscience.iop.org/1742-5468/2008/10/P10008/

[57] Csardi G, Nepusz T. The igraph software package for complex network research. InterJournal [Internet]. 2006; Complex Sy:1695. Available from: http://igraph.sf.net

[58] Veech JA (2014). The pairwise approach to analysing species co-occurrence. J Biogeogr.

[59] Witten IH, Frank E, Hall MA. Data mining: practical machine learning tools and techniques, third edition [internet]. Ann Phys (N Y). 2011. Available from: http://www.cs.waikato.ac.nz/~ml/weka/book.html%5Cnhttp://www.amazon.com/Data-Mining-Practical-Techniques-Management/dp/0123748569

[60] Vens C, Rosso M-N, Danchin EGJ. Identifying discriminative classification-based motifs in biological sequences. Bioinformatics [Internet]. 2011 [cited 2017 Dec 23];27:1231–1238. Available from: http://www.ncbi.nlm.nih.gov/pubmed/21372086. 10.1093/bioinformatics/btr110 21372086

[61] Yang J, Chen X, McDermaid A, Ma Q. DMINDA 2.0: integrated and systematic views of regulatory DNA motif identification and analyses. Bioinformatics. 2017; 10.1093/bioinformatics/btx223 28419194

[62] Wu B, Su S, Patil DP, Liu H, Gan J, Jaffrey SR, et al. Molecular basis for the specific and multivariate recognitions of RNA substrates by human hnRNPA2/B1. bioRxiv [Internet]. 2017; [cited 2017 Jun 24]; Available from: http://www.biorxiv.org/content/early/2017/06/01/144345

[63] Zhang X, Niu D, Carbonell A, Wang A, Lee A, Tun V, et al. ARGONAUTE PIWI domain and microRNA duplex structure regulate small RNA sorting in Arabidopsis. Nat. Commun. [Internet]. Nature Publishing Group; 2014 [cited 2017 Dec 23];5:5468. Available from: http://www.nature.com/doifinder/10.1038/ncomms6468 10.1038/ncomms6468 PMC4238042 25406978

[64] Klein M, Chandradoss SD, Depken M, Joo C. Why Argonaute is needed to make microRNA target search fast and reliable. Semin Cell Dev Biol [Internet] Academic Press; 2017 [cited 2017 Dec 23];65:20–28. Available from: https://www.sciencedirect.com/science/article/pii/S1084952116301434 10.1016/j.semcdb.2016.05.017 27235676

[65] Schirle NT, Sheu-Gruttadauria J, MacRae IJ. Structural basis for microRNA targeting. Science [Internet] American Association for the Advancement of Science; 2014 [cited 2017 Dec 23];346:608–613. Available from: http://www.ncbi.nlm.nih.gov/pubmed/25359968. 10.1126/science.1258040 PMC4313529 25359968

[66] Werfel S, Leierseder S, Ruprecht B, Kuster B, Engelhardt S. Preferential microRNA targeting revealed by in vivo competitive binding and differential Argonaute immunoprecipitation. Nucleic Acids Res [Internet] Oxford University Press; 2017 [cited 2017 Dec 23];45:10218–10228. Available from: http://academic.oup.com/nar/article/45/17/10218/4037351 10.1093/nar/gkx640 PMC5622317 28973447

