# Supplementary material for: A systematic approach to RNA-associated motif discovery
Source: BMC Genomics. 2018 Feb 14;19:146. doi: 10.1186/s12864-018-4528-x (PMC5813387; doi:10.1186/s12864-018-4528-x)
Supplement: Supplementary file 1 — Supplementary Figures and Tables. (PDF 1044 kb) [file 12864_2018_4528_MOESM1_ESM.pdf]

A Systematic Approach to RNA-Associated Motif Discovery

Tian Gao[1,‡], Jiang Shu[1,‡], Juan Cui[1,*]

[1]Systems Biology and Biomedical Informatics (SBBI) Laboratory, Department of Computer Science and Engineering, University of Nebraska-Lincoln, Lincoln, NE, 68588, USA

* To whom correspondence should be addressed. Tel: +1 402 472 5023; Fax: +1 402 472 7767; Email: jcui@unl.edu.

‡ These authors contributed equally to this work as first authors.

Supplementary Information

Supplementary Figures

Motif Length	Predicted Motif	Coverage (out of 30)	IC	Adj. *p*-value	
				RNA	miR
3	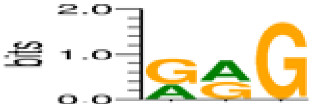	30	1.22	1.3E-04	2.6E-04
	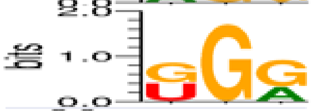	30	1.22	3.2E-04	2.0E-03
4	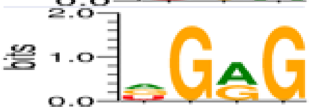	30	1.64	5.6E-06	3.7E-05
	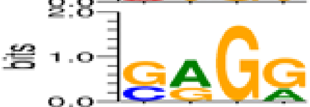	29	1.56	5.5E-05	6.8E-05
	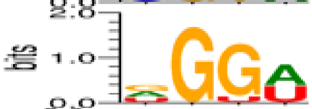	27	1.41	2.7E-03	4.1E-02
5	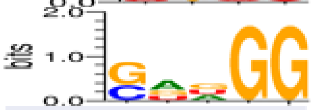	28	1.80	3.7E-06	1.7E-05
	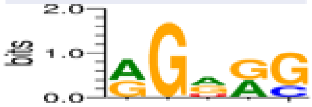	28	1.70	1.9E-05	1.7E-04

Figure S1. MDS^2-predicted motifs based on the 30 exosomal miRNAs reported in Villarroya-Beltri *et al.*

Motif Length	Predicted Motif	Coverage (out of 103)	IC	Adj. *p*-value	
				RNA	miR
3	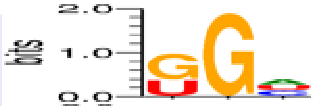	101	1.03	5.4E-03	1.0E+00
	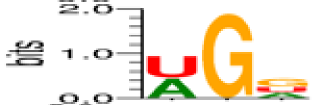	99	1.03	2.9E-03	9.1E-01
4	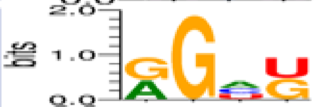	89	1.34	9.8E-06	1.2E-03
	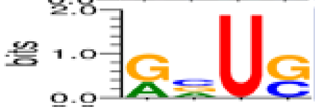	86	1.38	4.8E-05	7.0E-03
	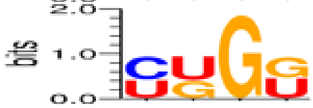	73	1.51	1.8E-06	8.0E-02

Figure S2. MDS[2]-predicted motifs based on the 103 exosomal miRNAs reported in Santangelo *et al.*

Motif Length	Predicted Motif	Coverage (out of 39)	IC	Adj. *P*-value	
				RNA	miRNA
3	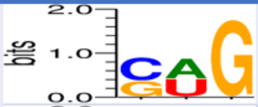	37	1.21	7.6E-03	1.0E+00
	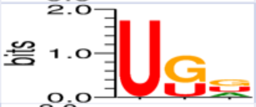	36	1.04	3.2E-02	1.0E+00
4	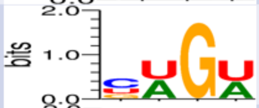	32	1.35	1.2E-03	1.5E-01
	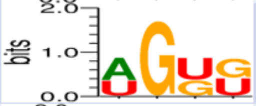	29	1.51	3.2E-04	3.7E-01
5	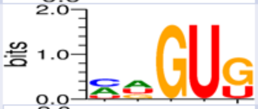	24	1.78	2.6E-05	1.2E-02
	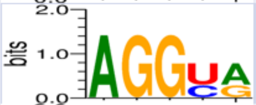	11	2.42	7.0E-04	7.3E-03
	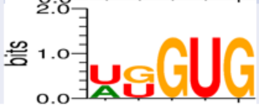	9	2.41	4.7E-03	3.0E-01

Figure S3. MDS^2-predicted motifs based on the 39 exosomal miRNAs in SW620 cell

Motif Length	Predicted Motif	Coverage (out of 112)	IC	Adj. *P*-value	
				RNA	miRNA
2	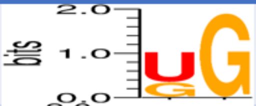	111	0.90	1.0E-04	1.6E-01
3	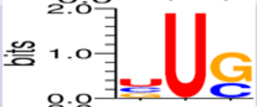	110	1.05	1.0E-04	5.9E-01
	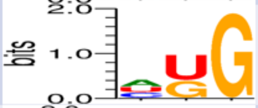	108	1.04	9.5E-03	1.0E+00
4	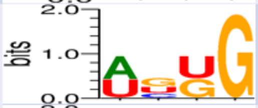	100	1.35	1.8E-07	1.6E-02
	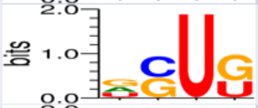	97	1.34	7.0E-08	1.7E-01
	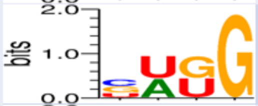	96	1.35	1.7E-07	7.2E-02
	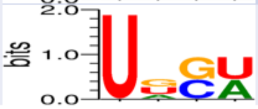	91	1.35	2.4E-06	7.0E-02

Figure S4. MDS[2]-predicted motifs based on the 112 exosomal miRNAs in SW620 cell

Motif Length	Predicted Motif	Coverage (out of 290)	IC	Adj. *P*-value
3	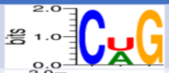	284	1.51	7.4E-03
4	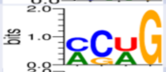	286	1.54	8.8E-03
	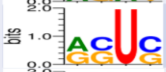	285	1.51	2.3E-03
	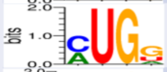	278	1.71	2.4E-05
5	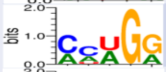	283	1.74	9.1E-06
	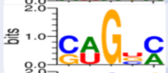	283	1.70	1.3E-05
	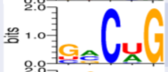	281	1.71	7.1E-04
	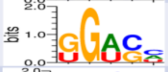	279	1.68	5.7E-05
6	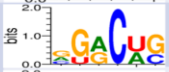	263	2.03	5.8E-16
	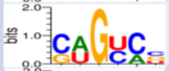	259	2.03	4.7E-16
	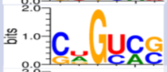	251	2.05	1.1E-09
	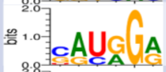	248	2.07	8.8E-09
	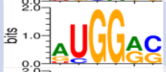	201	2.38	6.5E-11
	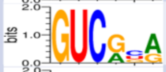	123	2.63	1.4E-09
	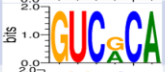	69	3.31	6.5E-14
7	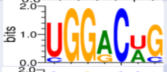	169	3.23	1.9E-40
	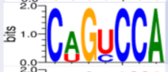	149	3.72	2.0E-38
	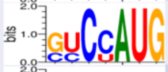	145	3.43	7.3E-36
	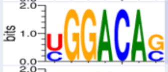	85	3.64	1.9E-24
	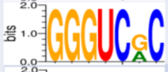	81	3.93	2.5E-21
	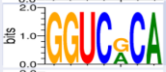	66	3.92	2.2E-19
	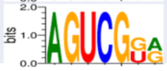	64	3.61	2.7E-18

Figure S5. MDS^2-predicted motifs based on the 290 exosomal mRNAs identified in our in-house bovine milk sequencing data

Motif Length	Predicted Motif	Coverage (out of 37)	IC	Adj. *P*-value	
				RNA	miRNA
3	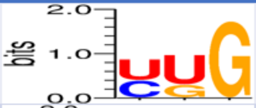	33	1.21	1.9E-02	1.0E+00
4	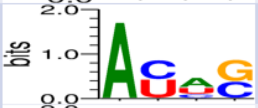	31	1.35	1.1E-02	1.2E-03
	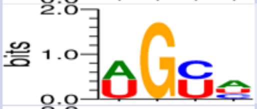	31	1.34	6.3E-03	2.9E-01
	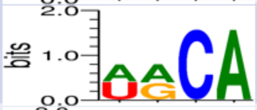	18	1.81	1.6E-02	6.8E-02
5	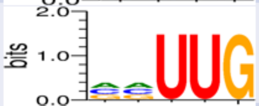	13	2.06	1.3E-03	1.4E-02
	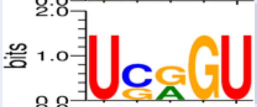	12	2.42	2.7E-05	9.0E-05
	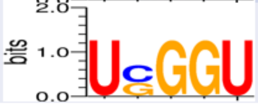	7	2.71	6.0E-04	5.7E-03

Figure S6. MDS[2]-predicted motifs based on the 37 exosomal microRNAs identified in our in-house bovine milk sequencing data

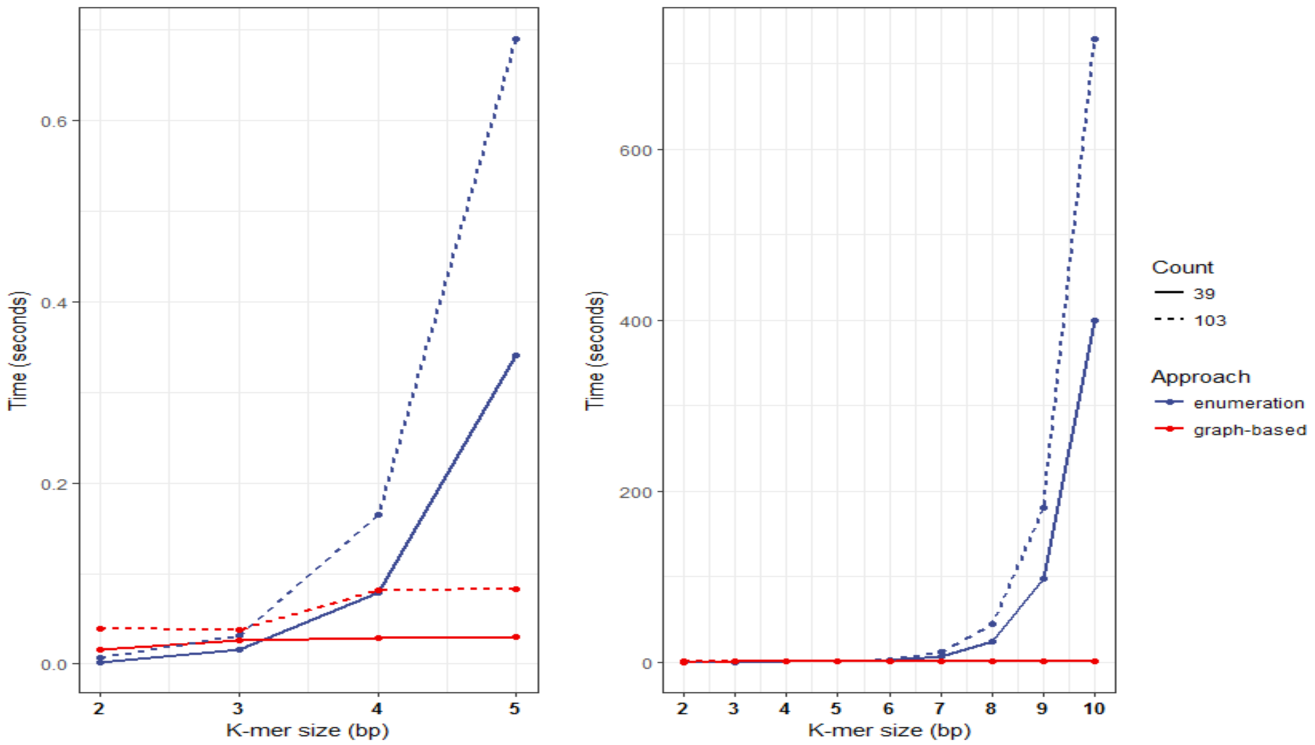

Figure S7. Searching time for significant k-mers of length 2-5 bps (left) and 2-10 bps (right) showing the performance of the current approach versus an enumeration-based approach on significant k-mer search. The results show that the dimer graph takes less time than the other as the k increases.

Supplementary Table

Table S1. Exosomal miRNAs in SW620

Datasets	miRNAs	Sequences
SW620-39	hsa-miR-499a-5p	UUAAGACUUGCAGUGAUGUUU
SW620-39	hsa-miR-23b-5p	UGGGUUCCUGGCAUGCUGAUUU
SW620-39	hsa-miR-323a-5p	AGGUGGUCCGUGGCGCGUUCGC
SW620-39	hsa-miR-890	UACUUGGAAAGGCAUCAGUUG
SW620-39	hsa-miR-488-5p	CCCAGAUAAUGGCACUCUCAA
SW620-39	hsa-miR-128-3p	UCACAGUGAACCGGUCUCUUU
SW620-39	hsa-miR-564	AGGCACGGUGUCAGCAGGC
SW620-39	hsa-miR-210-3p	CUGUGCGUGUGACAGCGGCUGA
SW620-39	hsa-miR-769-5p	UGAGACCUCUGGGUUCUGAGCU
SW620-39	hsa-miR-452-3p	CUCAUCUGCAAAGAAGUAAGUG
SW620-39	hsa-miR-21-5p	UAGCUUAUCAGACUGAUGUUGA
SW620-39	hsa-miR-411-3p	UAUGUAACACGGUCCACUAACC
SW620-39	hsa-miR-17-5p	CAAAGUGCUUACAGUGCAGGUAG
SW620-39	hsa-miR-138-5p	AGCUGGUGUUGUGAAUCAGGCCG
SW620-39	hsa-miR-95-3p	UUCAACGGGUAUUUAUUGAGCA
SW620-39	hsa-miR-24-1-5p	UGCCUACUGAGCUGAUAUCAGU
SW620-39	hsa-miR-17-3p	ACUGCAGUGAAGGCACUUGUAG
SW620-39	hsa-miR-302d-3p	UAAGUGCUUCCAUGUUUGAGUGU
SW620-39	hsa-miR-509-5p	UACUGCAGACAGUGGCAAUCA
SW620-39	hsa-miR-339-5p	UCCCUGUCCUCCAGGAGCUCACG
SW620-39	hsa-miR-185-5p	UGGAGAGAAAGGCAGUUCCUGA
SW620-39	hsa-miR-342-3p	UCUCACACAGAAAUCGCACCCGU
SW620-39	hsa-miR-758-3p	UUUGUGACCUGGUCCACUAACC
SW620-39	hsa-miR-326	CCUCUGGGCCCUUCCUCCAG
SW620-39	hsa-miR-483-3p	UCACUCCUCUCCUCCCGUCUU
SW620-39	hsa-miR-619-3p	GACCUGGACAUGUUUGUGCCCAGU
SW620-39	hsa-miR-595	GAAGUGUGCCGUGGUGUGUCU
SW620-39	hsa-miR-194-3p	CCAGUGGGGCUGCUGUUAUCUG
SW620-39	hsa-miR-587	UUUCCAUAGGUGAUGAGUCAC
SW620-39	hsa-miR-331-5p	CUAGGUAUGGUCCCAGGGAUCC
SW620-39	hsa-miR-603	CACACACUGCAAUUACUUUUGC
SW620-39	hsa-miR-383-5p	AGAUCAGAAGGUGAUUGUGGCU
SW620-39	hsa-miR-340-3p	UCCGUCUCAGUUACUUUAUAGC
SW620-39	hsa-miR-518e-5p	CUCUAGAGGGAAGCGCUUUCUG
SW620-39	hsa-miR-1224-5p	GUGAGGACUCGGGAGGUGG

SW620-39	hsa-miR-518b	CAAAGCGCUCCCCUUUAGAGGU
SW620-39	hsa-miR-620	AUGGAGAUAGAUAUAGAAAU
SW620-39	hsa-let-7a-5p	UGAGGUAGUAGGUUGUAUAGUU
SW620-39	hsa-miR-548d-5p	AAAAGUAAUUGUGGUUUUUGCC
SW620-112	hsa-miR-483-5p	AAGACGGGAGGAAAGAAGGGAG
SW620-112	hsa-miR-765	UGGAGGAGAAGGAAGGUGAUG
SW620-112	hsa-miR-301a	CAGUGCAAUAGUAUUGUCAAAGC
SW620-112	hsa-miR-1246	AAUGGAUUUUUGGAGCAGG
SW620-112	hsa-miR-630	AGUAUUCUGUACCAGGGAAGGU
SW620-112	hsa-miR-654-5p	UGGUGGGCCGCAGAACAUGUGC
SW620-112	hsa-miR-1275	GUGGGGGAGAGGCUGUC
SW620-112	hsa-miR-1268	CGGGCGUGGUGGUGGGGG
SW620-112	hsa-miR-30c-2-3p	CUGGGAGAAGGCUGUUUACUCU
SW620-112	hsa-miR-516b	AUCUGGAGGUAAGAAGCACUUU
SW620-112	hsa-miR-516a-5p	UUCUCGAGGAAAGAAGCACUUUC
SW620-112	hsa-miR-1182	GAGGGUCUUGGGAGGGAUGUGAC
SW620-112	hsa-miR-1207-5p	UGGCAGGGAGGCUGGGAGGGG
SW620-112	hsa-miR-638	AGGGAUCGCGGGCGGGUGGCGGCCU
SW620-112	hsa-miR-30c-1-3p	CUGGGAGAGGGUUGUUUACUCC
SW620-112	hsa-miR-609	AGGGUGUUUCUCUCAUCUCU
SW620-112	hsa-miR-451	AAACCGUUACCAUUACUGAGUU
SW620-112	hsa-miR-1207-3p	UCAGCUGGCCCUCAUUUC
SW620-112	hsa-miR-526b	CUCUUGAGGGAAGCACUUUCUGU
SW620-112	hsa-miR-1184	CCUGCAGCGACUUGAUGGCUUCC
SW620-112	hsa-miR-519c-3p	AAAGUGCAUCUUUUUAGAGGAU
SW620-112	hsa-miR-659	CUUGGUUCAGGGAGGGUCCCCA
SW620-112	hsa-miR-32-3p	CAAUUUAGUGUGUGUGAUAUUU
SW620-112	hsa-miR-1202	GUGCCAGCUGCAGUGGGGGAG
SW620-112	hsa-miR-1292	UGGGAACGGGUUCCGGCAGACGCUG
SW620-112	hsa-miR-146b-5p	UGAGAACUGAAUUCCAUAGGCU
SW620-112	hsa-miR-509-3p	UGAUUGGUACGUCUGUGGGUAG
SW620-112	hsa-miR-624	CACAAGGUAUUGGUAUUACCU
SW620-112	hsa-miR-616-5p	ACUCAAAACCCUUCAGUGACUU
SW620-112	hsa-miR-135a	UAUGGCUUUUUAUUCCUAUGUGA
SW620-112	hsa-miR-648	AAGUGUGCAGGGCACUGGU
SW620-112	hsa-miR-1284	UCUAUACAGACCCUGGCUUUUC
SW620-112	hsa-miR-512-3p	AAGUGCUGUCAUAGCUGAGGUC
SW620-112	hsa-miR-98	UGAGGUAGUAAGUUGUAUUGUU
SW620-112	hsa-miR-582-5p	UUACAGUUGUUCAACCAGUUACU

SW620-112	hsa-miR-29c	UAGCACCAUUUGAAAUCGGUUA
SW620-112	hsa-miR-518d-3p	CAAAGCGCUUCCCUUUGGAGC
SW620-112	hsa-miR-1228	UCACACCUGCCUCGCCCCCC
SW620-112	hsa-miR-30b-3p	CUGGGAGGUGGAUGUUUACUUC
SW620-112	hsa-miR-452	AACUGUUUGCAGAGGAAACUGA
SW620-112	hsa-miR-26b-3p	CCUGUUCUCCAUUACUUGGCUC
SW620-112	hsa-miR-367-5p	ACUGUUGCUAAUAUGCAACUCU
SW620-112	hsa-miR-548n	CAAAAGUAAUUGUGGAUUUUGU
SW620-112	hsa-miR-299-3p	UAUGUGGGAUGGUAAACCGCUU
SW620-112	hsa-miR-454	UAGUGCAAUAUUGCUUAUAGGGU
SW620-112	hsa-miR-376c	AACAUAGAGGAAAUUCCACGU
SW620-112	hsa-miR-1225-5p	GUGGGUACGGCCCAGUGGGGGG
SW620-112	hsa-miR-767-5p	UGCACCAUGGUUGUCUGAGCAUG
SW620-112	hsa-miR-1909	CGCAGGGGCCGGGUGCUCACCG
SW620-112	hsa-miR-200b-5p	CAUCUUACUGGGCAGCAUUGGA
SW620-112	hsa-miR-337-3p	CUCCUAUAUGAUGCCUUUCUUC
SW620-112	hsa-miR-1282	UCGUUUGCCUUUUUCUGCUU
SW620-112	hsa-let-7b	UGAGGUAGUAGGUUGUGUGGUU
SW620-112	hsa-miR-1911	UGAGUACCGCCAUGUCUGUUGGG
SW620-112	hsa-miR-940	AAGGCAGGGCCCCCGCUCCCC
SW620-112	hsa-miR-566	GGGCGCCUGUGAUCCCAAC
SW620-112	hsa-miR-617	AGACUUCCCAUUUGAAGGUGGC
SW620-112	hsa-miR-520e	AAAGUGCUUCCUUUUUGAGGG
SW620-112	hsa-miR-517b	AUCGUGCAUCCCUUUAGAGUGU
SW620-112	hsa-miR-320c	AAAAGCUGGGUUGAGAGGGU
SW620-112	hsa-miR-550	UGUCUUACUCCCUCAGGCACAU
SW620-112	hsa-miR-624-5p	UAGUACCAGUACCUUGUGUUCA
SW620-112	hsa-miR-188-5p	CAUCCCUUGCAUGGUGGAGGG
SW620-112	hsa-miR-320d	AAAAGCUGGGUUGAGAGGA
SW620-112	hsa-miR-361-3p	UCCCCCAGGUGUGAUUCUGAUUU
SW620-112	hsa-miR-498	UUUCAAGCCAGGGGGCGUUUUUC
SW620-112	hsa-miR-885-3p	AGGCAGCGGGGUGUAGUGGAUA
SW620-112	hsa-miR-190	UGAUAUGUUUGAUAUAUUAGGU
SW620-112	hsa-miR-181a-3p	ACCAUCGACCGUUGAUUGUACC
SW620-112	hsa-miR-192	CUGACCUAUGAAUUGACAGCC
SW620-112	hsa-miR-1183	CACUGUAGGUGAUGGUGAGAGUGGGCA
SW620-112	hsa-miR-1306	ACGUUGGCUCUGGUGGUG
SW620-112	hsa-miR-15a	UAGCAGCACAUAAUGGUUUGUG
SW620-112	hsa-miR-557	GUUUGCACGGGUGGGCCUUGUCU

SW620-112	hsa-miR-876-5p	UGGAUUUCUUUGUGAAUCACCA
SW620-112	hsa-miR-135b	UAUGGCUUUUCAUUCCUAUGUGA
SW620-112	hsa-let-7e-3p	CUAUACGGCCUCCUAGCUUUCC
SW620-112	hsa-miR-186-3p	GCCCAAAGGUGAAUUUUUUGGG
SW620-112	hsa-miR-551b	GCGACCCAUACUUGGUUUCAG
SW620-112	hsa-miR-671-5p	AGGAAGCCCUGGAGGGGCUGGAG
SW620-112	hsa-miR-34a-3p	CAAUCAGCAAGUAUACUGCCCU
SW620-112	hsa-miR-92a	UAUUGCACUUGUCCCGGCCUGU
SW620-112	hsa-miR-1915	CCCCAGGGCGACGCGGCGGG
SW620-112	hsa-miR-1279	UCAUAUUGCUUCUUUCU
SW620-112	hsa-miR-665	ACCAGGAGGCUGAGGCCCCU
SW620-112	hsa-miR-1272	GAUGAUGAUGGCAGCAAAUUCUGAAA
SW620-112	hsa-miR-572	GUCCGCUCGGCGGUGGCCCA
SW620-112	hsa-miR-135a-3p	UAUAGGGAUUGGAGCCGUGGCG
SW620-112	hsa-miR-320b	AAAAGCUGGGUUGAGAGGGCAA
SW620-112	hsa-miR-455-3p	GCAGUCCAUGGGCAUAUACAC
SW620-112	hsa-miR-575	GAGCCAGUUGGACAGGAGC
SW620-112	hsa-miR-512-5p	CACUCAGCCUUGAGGGCACUUUC
SW620-112	hsa-miR-1197	UAGGACACAUGGUCUACUUCU
SW620-112	hsa-miR-892b	CACUGGCUCCUUUCUGGGUAGA
SW620-112	hsa-miR-181b	AACAUUCAUUGCUGUCGGUGGGU
SW620-112	hsa-miR-612	GCUGGGCAGGGCUUCUGAGCUCCUU
SW620-112	hsa-miR-125b	UCCCUGAGACCCUAACUUGUGA
SW620-112	hsa-miR-576-3p	AAGAUGUGGAAAAAUUGGAAUC
SW620-112	hsa-miR-548c-3p	CAAAAAUCUCAAUUACUUUUGC
SW620-112	hsa-miR-18b	UAAGGUGCAUCUAGUGCAGUUAG
SW620-112	hsa-miR-517-5p	CCUCUAGAUGGAAGCACUGUCU
SW620-112	hsa-miR-320a	AAAAGCUGGGUUGAGAGGGCGA
SW620-112	hsa-miR-542-5p	UCGGGGAUCAUCAUGUCACGAGA
SW620-112	hsa-miR-877-3p	UCCUCUUCUCCCUCCUCCCAG
SW620-112	hsa-miR-424	CAGCAGCAAUUCAUGUUUUGAA
SW620-112	hsa-miR-577	UAGAUAAAAUAUUGGUACCUG
SW620-112	hsa-miR-506	UAAGGCACCCUUCUGAGUAGA
SW620-112	hsa-miR-130b	CAGUGCAAUGAUGAAAGGGCAU
SW620-112	hsa-miR-574-3p	CACGCUCAUGCACACACCCACA
SW620-112	hsa-miR-218-2-3p	CAUGGUUCUGUCAAGCACCGCG
SW620-112	hsa-miR-191	CAACGGAAUCCCAAAAGCAGCUG
SW620-112	hsa-miR-493	UGAAGGUCUACUGUGUGCCAGG
